# Cerebrovascular reactivity mapping using breath-hold BOLD-fMRI: Comparison of signal models combined with voxelwise lag optimization

**DOI:** 10.1162/IMAG.a.80

**Published:** 2025-07-14

**Authors:** Catarina Domingos, Inês Esteves, Ana R. Fouto, Amparo Ruiz-Tagle, Gina Caetano, César Caballero-Gaudes, Patrícia Figueiredo

**Affiliations:** Institute for Systems and Robotics - Lisboa and Department of Bioengineering, Instituto Superior Técnico, Universidade de Lisboa, Lisbon, Portugal; Basque Center on Cognition Brain and Language, San Sebastián-Donostia, Spain; Ikerbasque Basque Foundation for Science, Bilbao, Spain

**Keywords:** BOLD-fMRI, breath-hold, cerebrovascular reactivity, PetCO2, hemodynamic response function, lag optimization

## Abstract

Cerebrovascular reactivity (CVR) can be mapped noninvasively using blood oxygenation level dependent (BOLD) fMRI during a breath-hold (BH) task. Previous studies showed that the BH BOLD response is best modeled as the convolution of the partial pressure of end-tidal CO2 (PetCO2) with a canonical hemodynamic response function (HRF). However, previous model comparisons employed a global bulk time lag, which is now well accepted to provide only a rough approximation of the heterogeneous distribution of response latencies across the brain. Here, we investigate the best modeling approach for mapping CVR based on BH BOLD-fMRI data, when using a lagged general linear model approach for voxelwise lag optimization. In a group of 14 healthy participants, we compared two types of regressors (PetCO2 and Block), and three convolution models (no convolution; convolution with a single gamma HRF; and convolution with a double gamma HRF), as well as a range of HRF delays and dispersions (for models with convolution). Convolution with a single gamma HRF yielded the greatest CVR values in PetCO2 models, while a double gamma HRF performed better for block models. Although PetCO2-based regressors generally outperformed block-based regressors, as expected, the latter may be an appropriate alternative in cases of poor CO_2_ recordings. Overall, our results support the use of specific modeling approaches for CVR mapping based on end-expiration BH BOLD-fMRI, including the voxelwise optimization of the lag.

## 1 Introduction

Cerebrovascular reactivity (CVR) reflects the ability of brain vessels to alter their caliber and adjust cerebral blood flow (CBF) in response to vasoactive stimuli, as for instance carbon dioxide (CO_2_) (Chen & Fierstra, 2022). CVR is usually evaluated by manipulating respiratory gases to induce hypercapnia or hypocapnia, either through external gas inhalation modulating the CO_2_ content in the inspired gas (Liu et al., 2017), or through the execution of respiratory tasks such as breath-holding (BH) (K. Chen et al., 2021; Urback et al., 2017) or deep breathing (Bright et al., 2009; Sousa et al., 2014). BH tasks have been frequently used for CVR assessment because they do not require external gas manipulation and are, therefore, less uncomfortable and easier to implement than gas inhalation procedures (Pinto et al., 2021). Although BH challenges also present some drawbacks, depending on the subject’s compliance with the task instructions, potentially yielding higher variability and often inducing task-related head movement, carefully designed paradigms explicitly targeting end-expiration BH may be used to mitigate these (Pinto et al., 2021; Urback et al., 2017). For mapping CVR with good spatial resolution, functional MRI (fMRI) can be used by relying on blood oxygenation level-dependent (BOLD) changes as a surrogate of CBF changes in response to the vasoactive stimulation (Pinto et al., 2021).

The BOLD response to BH is commonly modeled as the convolution of the partial pressure of end-tidal CO2 (PetCO2) with a hemodynamic response function (HRF) in a general linear modeling (GLM) framework (Birn et al., 2008). The canonical HRF, consisting of a double gamma function with a time to peak delay of 6 s (Glover, 1999), is most used. However, other HRF shapes and delays have been considered too, including single gamma functions (Sleight et al., 2021; Vogt et al., 2011). PetCO2 recordings have also been used directly as a regressor without convolution with an HRF (Hou et al., 2020; Liu et al., 2020; Murphy et al., 2011). In the absence of a reliable PetCO2 signal, block paradigms depicting the BH periods may be considered (Birn et al., 2008; Biswal et al., 2007; Bright & Murphy, 2013; Kastrup et al., 1999; Murphy et al., 2011). Alternatively, Fourier models have also been proposed to describe the periodic response pattern to a sequence of BH periods based on combinations of sines and cosines (Lipp et al., 2015; Murphy et al., 2011; Pinto et al., 2016; van Niftrik et al., 2016). More recently, Zvolanek et al. (2023) further showed that models based on the respiration volume per time and average gray matter signal provided comparable CVR maps to those based on PetCO2 recordings.

One critical consideration when modeling CVR BOLD signals is the latency of the response to the vasoactive stimuli. In earlier studies, a global time lag (Bright & Murphy, 2013; Bright et al., 2009) was typically estimated based on the cross-correlation between the PetCO2 signal and the average gray matter BOLD signal. However, the observation of a significant range of latencies across the brain has more recently motivated the estimation of regional time lags, ultimately voxelwise using a lagged GLM approach (K. Chen et al., 2021; Moia et al., 2020; Stickland et al., 2021; Zvolanek et al., 2023). The only previous study comparing different modeling approaches for BOLD responses to BH tasks (Murphy et al., 2011) did not explicitly consider voxelwise lag optimization, although that is implicit to the sine-cosine model. For both PetCO2 and block models, large improvements [in explained variance] were obtained when adding a temporal derivative, implying that the dynamics of the BOLD response to BH was not fully explained by the canonical HRF. This suggests that the model of the BOLD response could benefit from further optimization, particularly regarding its timing, that is, its temporal delay and dispersion. Furthermore, given the close interaction with the response lag, the best signal model may differ when performing voxelwise lag optimization.

In summary, the current “golden standard” of PetCO2 convolved with the canonical HRF combined with its temporal derivative has been shown to outperform alternative models (Murphy et al., 2011). However, an investigation considering different HRFs with different shapes and delays in combination with voxelwise lag optimization has not been reported. Here, we investigate the optimal modeling approach for mapping CVR with voxelwise lag optimization in end-expiration BH BOLD-fMRI data acquired from 14 healthy participants in a main dataset (IST dataset) and validated in 9 subjects from a public dataset (EuskalIBUR dataset). We compared voxelwise lagged GLM considering: two types of regressors (PetCO2 and Block), three convolution models (no convolution; convolution with a single gamma HRF; and convolution with a double gamma HRF), and a range of HRF delays and dispersions.

## 2 Methods

### 2.1 Data acquisition

One main (private) dataset was first analyzed (N = 14 subjects)—IST dataset. The analysis was then reproduced on a second (public) independent dataset (N = 9 subjects)—EuskalIBUR dataset for assessing the generalizability of the results.

#### 2.1.1 IST dataset

A group of 17 healthy women (31 ± 8 years) were recruited as a control group in the scope of a study on menstrual migraine, with an MRI protocol encompassing several sequences acquired in multiple sessions along the migraine and menstrual cycles (Domingos et al., 2023; Fouto et al., 2024). The study was approved by the *Comissão de Ética para a Investigação Clínica of Hospital da Luz, Lisbon*. This study was carried out in accordance with the Declaration of Helsinki, and all subjects provided written informed consent. BH BOLD fMRI data were acquired in a subgroup of 14 healthy controls during the peri-menstrual phase, which are analyzed in this manuscript.

Volunteers were scanned on a 3T Siemens Vida MRI system (Siemens, Erlangen, Germany) using a head 64-channel receive radiofrequency (RF) coil. BOLD-fMRI data were acquired using a gradient-echo 2D-EPI sequence (TR/TE = 1260/30 ms, SMS acceleration factor = 3, in-plane GRAPPA acceleration factor = 2, echo spacing = 0.31 ms, EPI factor = 100, PE direction = A-P, voxel size = 2.20 mm isotropic, in-plane FOV = 220 × 220 mm^2^, total number of slices = 60). A 3D gradient-echo fieldmap (TE1/TE2 = 4.92/7.38 ms, voxel size = 3.4 × 3.4 × 3.0 mm^3^, FOV = 220 × 220 mm^2^, number of slices 45) and a T1-weighted structural image (MPRAGE, TR/TE = 2300.00/2.98 ms, voxel size = 1.00 mm isotropic, FOV = 256 × 240 mm^2^, number of slices = 176) were also acquired.

**Fig. 1.**
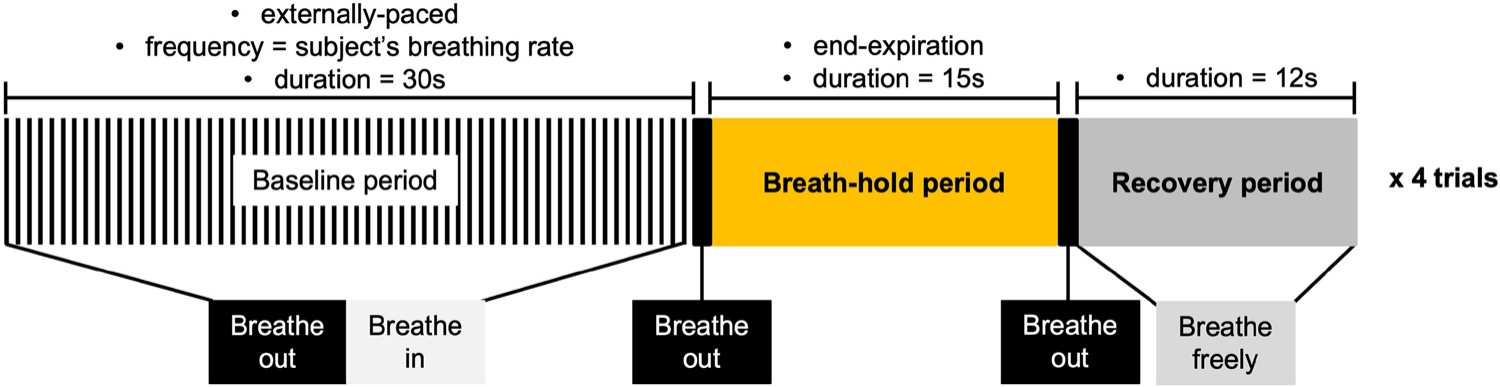
Illustration of the breath-hold (BH) task paradigm, with the respective parameters. Each BH was performed following an expiration, and it was followed by an exhalation and a free breathing recovery period. The baseline consisted in cued breathing at the subject’s breathing rate (as assessed in a calibration task before scanning). Adapted from Pinto et al. (2021).

The BH task paradigm is illustrated in Figure 1, consisting of four trials of 15 s of BH with expiration before and after, alternated with 30 s cued normal breathing, leading to a total acquisition time of 269 s and, consequently, 213 volumes. During the BOLD imaging of the BH task, the expired CO_2_ was sampled through a nasal cannula and measured using a Medlab CAP10 capnograph. Subjects were specifically instructed to breathe solely through their nose. To enhance compliance with the task and respective instructions, the subjects performed a practice session before entering the scanner. The capnograph’s frequency rate ranged from 1/3 Hz to 1/6 Hz, which was adjusted based on the patient’s breathing rate, as assessed during the preparation outside the scanner.

#### 2.1.2 EuskalIBUR dataset: cross-validation

A publicly available dataset acquired at the BCBL, San Sebastián - Donostia, Spain (https://openneuro.org/datasets/ds003192/versions/1.0.1) was used. In brief, this dataset includes 10 sessions of breath-holding task acquired in 9 healthy volunteers (24–40 years, 5F) on a 3T Siemens PrismaFit scanner (Siemens, Erlangen, Germany) using a head 64-channel receive RF coil. For this study, we selected only one session per individual based on the task performance and quality of end-tidal CO2 recordings (session 09 for subject 001, session 06 for subject 002 e 004, session 03 for subject 003 e 008, session 01 for subject 006, session 09 for subject 007 e 009, session 04 for subject 010). BOLD-fMRI data were acquired using a multiband multi-echo gradient-echo EPI sequence (TR = 1500 ms, TE = 10.6/28.69/46.78/64.87/82.96 ms, MB acceleration factor = 4, in-plane GRAPPA acceleration factor = 2, echo spacing = 0.31 ms, EPI factor = 100, PE direction = A-P, voxel size = 2.4 × 2.4 × 3 mm^3^, in-plane FOV = 211 × 211 mm^2^, total number of slices = 52). See Moia et al. (2021) for more details.

The BH task paradigm consisted of eight trials each comprising four paced breathing cycles of 6 s each, a BH of 20 s, an exhalation of 3 s, and 11 s of “recovery” breathing (unpaced) (i.e., total trial duration of 58 s). A resting period of 30 s was appended before the first and after the last BH trial. During the BOLD imaging of the BH task, the expired CO_2_ and O_2_ was monitored using a nasal cannula and measured using an AD Instruments gas analyzer connected to a BIOPAC MP150 physiological monitoring unit (Moia et al., 2021).

The preprocessing and subsequent analysis was conducted as described in sections 2.3 and 2.4, with the only difference that these data included T2*-weighted based optimal combination of the echoes.

### 2.2 CO_2_ data analysis

**Fig. 2.**
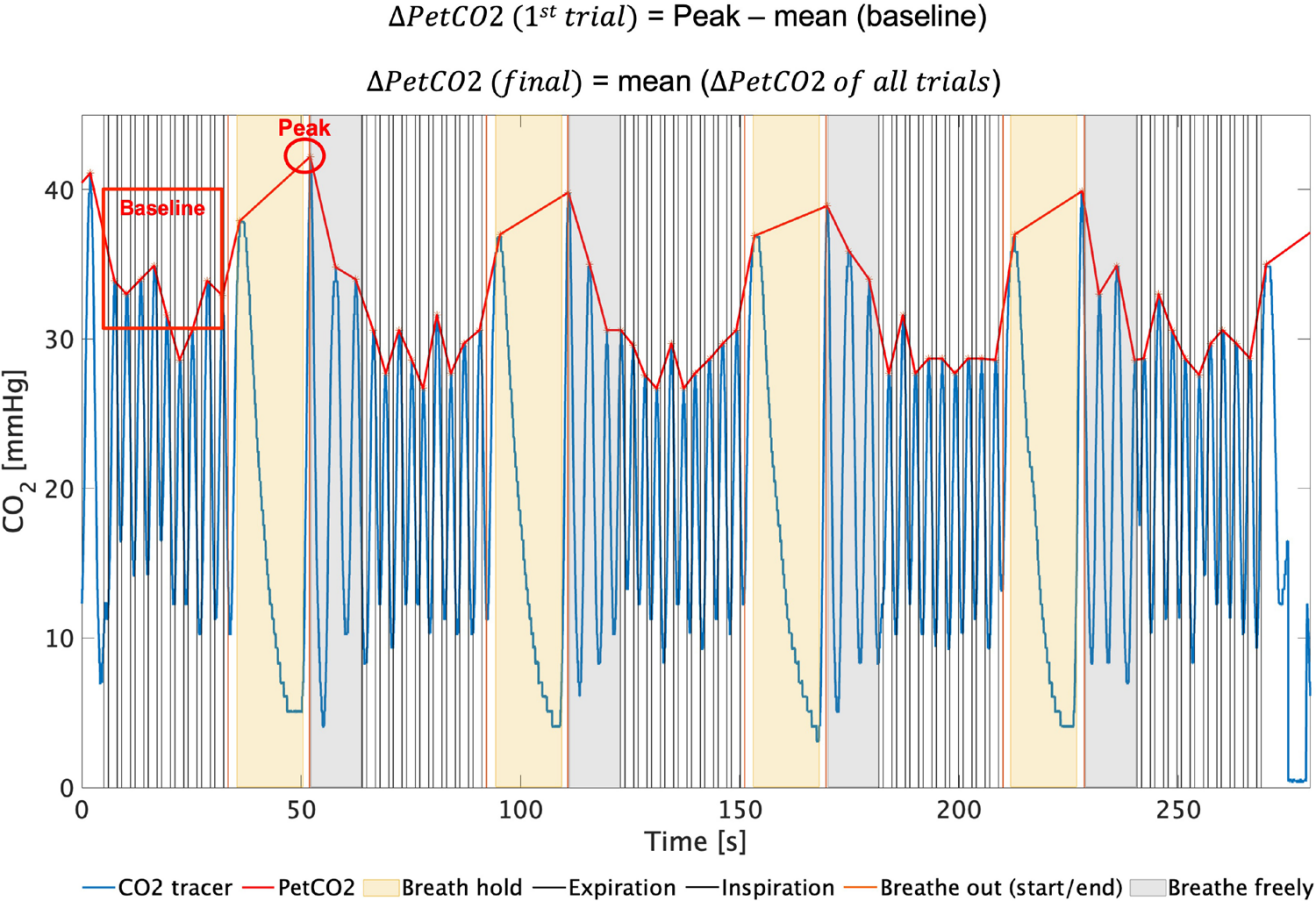
Illustration of the ∆PetCO2 calculation: CO_2_ signal for one representative subject, overlaid on the BH paradigm.

The CO_2_ signal measured by the capnograph was analyzed using MATLAB in-house code (version: 2016b, www.mathworks.com) to retrieve a PetCO2 trace, by peak detection, interpolation using a piecewise cubic interpolation (which produces the same results as the linear interpolation with an average correlation across subjects between the PetCO2 signal interpolated using a linear interpolation and using a cubic interpolation equals to 0.998 ± 0.0008) to a sampling rate equal to 0.027 Hz and application of a detrending filter with a cutoff at 100 s (to remove signal drifts, while preserving the signal of interest, since the respiratory rate is always higher than 0.15 Hz). Each trace was corrected to account for the time delay of the tube connecting the nasal cannula (inside the scanner) to the capnograph (outside the scanner). For each subject, the PetCO2 change between BH and baseline (ΔPetCO2 in mmHg) was computed by taking the first PetCO2 peak after each BH period and subtracting the average PetCO2 across the previous baseline period, and then averaging across the four BH periods, as illustrated in Figure 2.

### 2.3 Image analysis

**Fig. 3.**
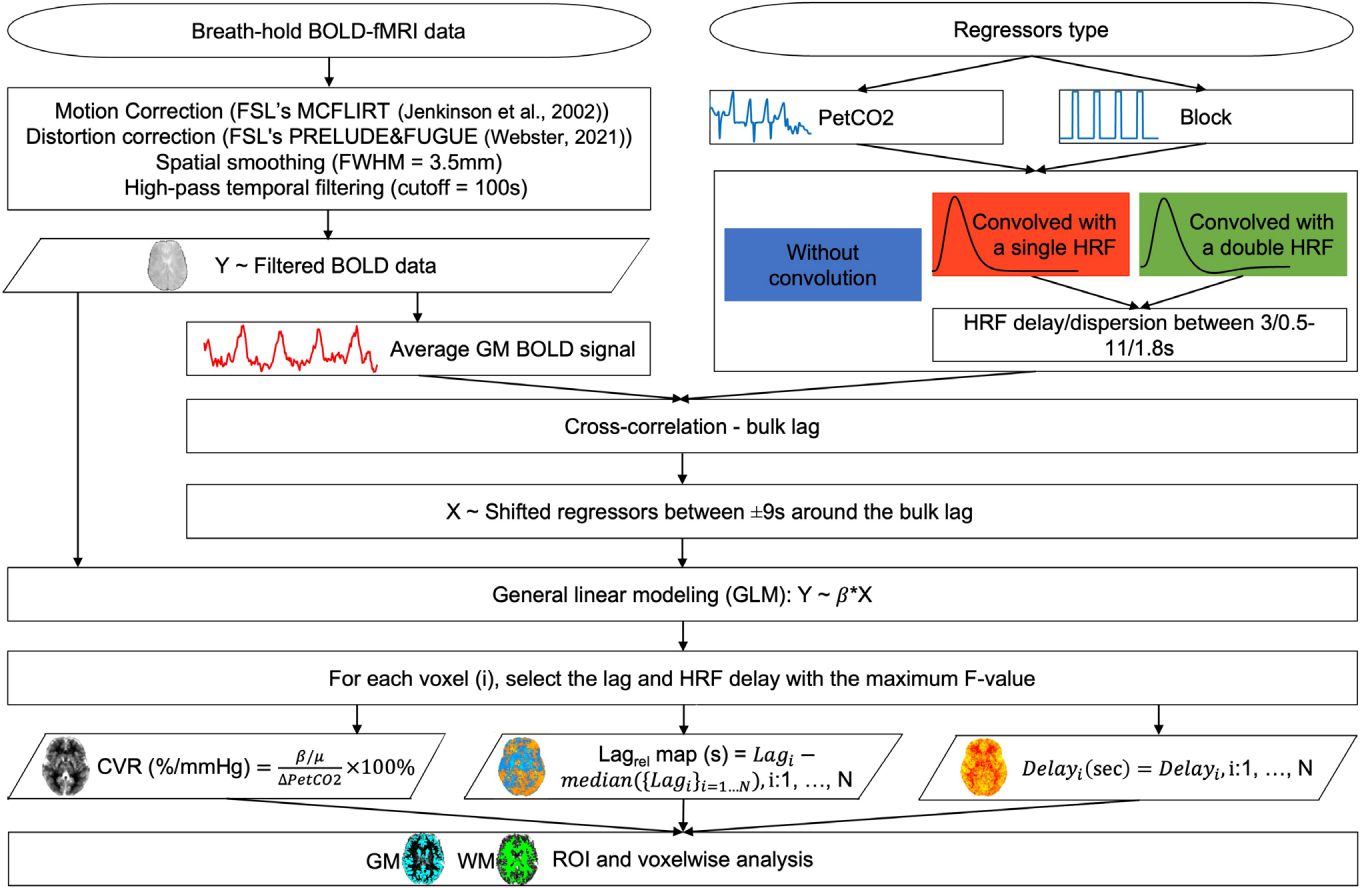
Breath-hold BOLD-fMRI data analysis pipeline, including the different models tested.

The full image analysis pipeline is displayed in Figure 3.

#### 2.3.1 Image preprocessing

Image analysis was performed using FSL tools (version: 6.0.0, fsl.fmrib.ox.ac.uk) (Jenkinson et al., 2012), particularly FSL’s FEAT toolbox. fMRI data preprocessing included: motion correction, distortion correction based on the fieldmap (Webster, 2021), spatial smoothing (FWHM = 3.5 mm), and detrending temporal filtering (cutoff = 100 s, to remove low-frequency artifacts, such as scanner drifts, without removing the signal of interest frequency ~0.02 Hz). Functional images were registered to the T1-weighted structural image using an affine transformation (Jenkinson et al., 2002), and to the MNI standard space using a nonlinear transformation (Andersson et al., 2010). The T1-weighted structural image was segmented into gray matter (GM), white matter (WM), and cerebrospinal fluid (CSF), and GM and WM masks were obtained by thresholding the respective partial volume estimate (PVE) maps at 50%, and subsequently registered to the functional space.

#### 2.3.2 Definition of BOLD signal models

To perform the GLM analysis, we considered a total of 6 BOLD signal models by combining the following factors: i) regressor type: PetCO2 or Block; and ii) convolution model: without convolution (WoC), convolved with a single gamma HRF (CSg), or convolved with a double gamma HRF (CDb). For CSg and CDb, we further optimized the HRF delay and dispersion coupling both parameters. Specifically, we consider HRF delays between 3 and 11 s in steps of 1 s each coupled with HRF dispersions between 3/6 and 11/6 s in steps of 1/6 s. Thus, a total of 9 HRF delays/dispersions were tested for each model with convolution.

##### 2.3.2.1 HRFs tested

**Fig. 4.**
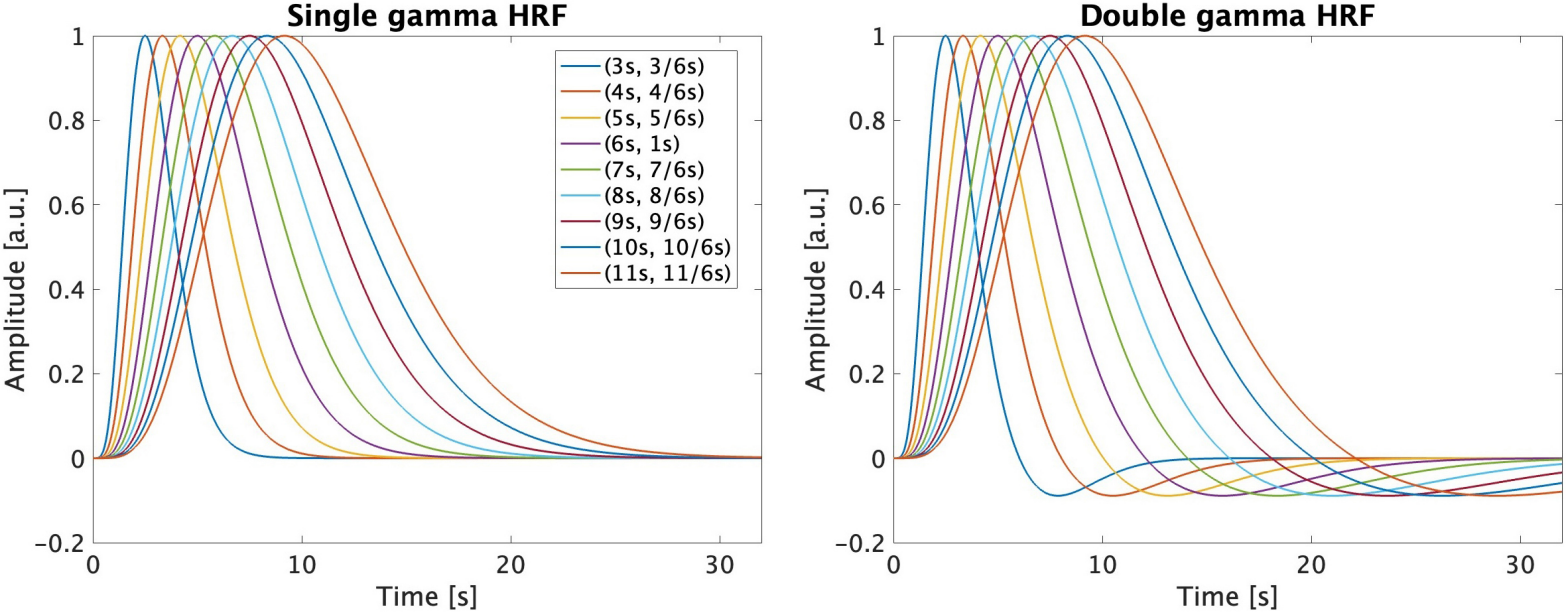
Hemodynamic response functions (HRFs) considered in the analysis: single-gamma (left) and double-gamma (right), for a range of delays/dispersions.

The HRFs were defined as a single gamma function (single-gamma), representing a positive response only, or a combination of two gamma functions (double-gamma), one representing a main positive peak and the other a subsequent smaller undershoot, according to equations 1.1 and 1.2, as implemented in the *spm_hrf* function of the MATLAB toolbox SPM12 (Ashburner et al., 2019):

$$HRF_{single}=spm\_Gpdf\left(u,\frac{p\left(1\right)}{p\left(3\right)},\frac{dt}{p\left(3\right)}\right)$$ (1.1)

$$\begin{array}{l} HRF_{double}=spm\_Gpdf\left(u,\frac{p\left(1\right)}{p\left(3\right)},\frac{dt}{p\left(3\right)}\right)\\ \;\;\;\;\;\;\;\;\;\;\;\;\;\;\;\;\;\;\;\;\;\;\;\;\;\;\;\;\;\;\;-\;spm\_Gpdf\frac{\left(u,\frac{p\left(2\right)}{p\left(4\right)},\frac{dt}{p\left(3\right)}\right)}{p\left(5\right)} \end{array}$$ (1.2)

where the *spm_Gpdf* function generates a probability density function (PDF) of the Gamma distribution with the parameters p(1) to p(5), as described in Table 1, along with the respective values tested here. While the HRF delay p(1) was varied between 3 and 11 s, the HRF dispersion p(3) was scaled as p(3) = p(1)/6. In the case of the double gamma, the delay and dispersion of the undershoot, p(2) and p(4), were adjusted to preserve the overall shape of the HRF. All the HRFs considered in the analysis are shown in Figure 4.

**Table 1.** Description of the HRF parameters as defined in Equations 1.1 and 1.2 (according to the SPM function spm_hrf).

Hemodynamic response function parameters									
Parameters	Values tested (s)								
p(1) = time to positive peak (relative to onset)	3	4	5	**6**	7	8	9	10	11
p(2) = time to undershoot (relative to onset)	8	32/3	40/3	**16**	56/3	64/3	24	80/3	88/3
p(3) = dispersion of positive peak	3/6	4/6	5/6	**1**	7/6	8/6	9/6	10/6	11/6
p(4) = dispersion of undershoot	3/6	4/6	5/6	**1**	7/6	8/6	9/6	10/6	11/6
p(5) = amplitude ratio between positive peak and undershoot	6	6	6	**6**	6	6	6	6	6

The canonical HRF parameters are highlighted in bold. The different HRF delays, p(1), and dispersions, p(3), tested are shown; the dispersion was scaled by the delay: p(3) = p(1)/6. Parameters p(2), p(4), and p(5) pertain only to the double gamma HRF. The delay and dispersion of the undershoot, p(2) and p(4), were adjusted to preserve the overall shape of the HRF.

##### 2.3.2.2 Response lags tested

For each subject and each model, the bulk time lag between the BH and the BOLD response was estimated as the lag that maximized the cross-correlation between the GM average BOLD time series and the model regressor (Moia et al., 2021; Stickland et al., 2021; Zvolanek et al., 2023). A limited range of lags between -15 and +15 s was considered, to avoid shifting the regressor by multiple cycles. The regressor was then shifted between -9 and +9 s (Moia et al., 2021; Stickland et al., 2021; Zvolanek et al., 2023) in increments of 1 s around the bulk lag, resulting in a total of 19 lags per subject and model. Other studies (Moia et al., 2021; Stickland et al., 2021; Zvolanek et al., 2023) used smaller shifts of 0.3 s, even though these shifts are only required to provide higher temporal resolution. A preliminary test on our data showed no significant difference in CVR values between applying a shift of 1 s and 0.3 s.

#### 2.3.3 Voxelwise GLM analysis

For each regressor type, convolution model, HRF delay/dispersion (for the models with convolution), and lag, a voxelwise GLM analysis of the BOLD-fMRI data was performed, including the regressor of interest (normalized between 0 and 1) as well as a total of 24 extended motion parameters (3 rotations, 3 translations, their 6 temporal derivatives, and the 12 squares of the above) and the motion outliers (identified based on the root mean square difference between each volume and the reference volume, the middle one; values beyond 1.5 times the 75^th^ InterQuartile range are considered outliers, resulting in 7 ± 4 volumes identified as outliers on average across subjects) as confounds, following the standard processing pipeline recommended for cleaning BOLD-fMRI data (Caballero-Gaudes & Reynolds, 2017; Friston et al., 1996), which is also used by other studies in the analysis of BOLD-fMRI CVR data (Bhogal, 2021; Moia et al., 2020). Here, pre-whitening was also used as recommended by FSL’s documentation (Jenkinson et al., 2012).

##### 2.3.3.1 Collinearity effects

Due to the high number of regressors in each GLM, we, nevertheless, evaluated the potential effects of the multicollinearity between model regressors (due to the intrinsic collinearity among the 24 motion parameters considered), by comparing the effect size required to estimate the BOLD response to PetCO2 changes when using only the standard 6 motion parameters or the extended 24 motion parameters. We found that the values increased only from 1.8 ± 0.2 to 1.9 ± 0.2% on average (a non-significant difference as assessed by a t-test, p > 0.05), which indicates that the added multicollinearity did not adversely affect our model fitting. Consistently, we also found relatively low correlation values (-0.2 to 0.2) between the PetCO2 regressor and the 24 motion parameters, as illustrated in Supplementary Figure S1.

##### 2.3.3.2 Lag optimization

The lag was optimized voxelwise, for each model and subject, following previous guidelines in the literature (Moia et al., 2020, 2021; Stickland et al., 2021; Zvolanek et al., 2023). For each voxel, the lag that yielded the regressor of interest with the highest F-value was selected as the one explaining the most signal variance (Murphy et al., 2011; Pinto et al., 2021). Since we restrict our model space to lags that we consider physiological meaningful, the maximum explained variance is an appropriate criterion for model selection. Furthermore, we evaluate the F-value of the partial model consisting of the regressor of interest and not the F-value of the full model (which includes also motion parameters and outliers).

##### 2.3.3.3 HRF delay/dispersion optimization

We also considered the voxelwise optimization of the HRF delay/dispersion, together with the lag, since this may potentially vary across the brain. However, an initial analysis revealed the inability to achieve an accurate estimation. The results are presented in Supplementary Figure S2, showing the distributions across subjects of the optimal values of HRF delay in GM and WM, for PetCO2 and Block regressors and the two convolution methods, considering optimization of both Lag_rel_ and HRF delay. The distributions show a saturation of the HRF delays at the extreme values, indicating a bias and suggesting that a good estimate could not be achieved. This is likely due to the strong interaction between the HRF delay and the lag, which limits the ability to estimate both parameters accurately given the SNR of the data. As a result, we opted not to undertake the voxelwise optimization of the HRF delay, and instead optimized this for the whole brain through an ROI analysis (see section 3.3). Because the HRF dispersion is directly related with the HRF delay (by design of the tested HRFs), the selection of an HRF delay implies the selection of the corresponding HRF dispersion (and this is not explicitly tested).

##### 2.3.3.4 Derivation of CVR and optimal relative lag maps

For each voxel i, the corresponding CVR_i_ value was obtained by dividing the parameter estimate, $\beta_{\text{i}}$, of the regressor of interest by the mean BOLD signal over time, $\mu_{\text{i}}$ and the subject’s mean ΔPetCO2 value:

$$CVR_{\text{i}}\left(\frac{\%}{mmHg}\right)=\frac{\frac{\beta_{\text{i}}}{\mu_{\text{i}}}}{\Delta PetCO2}\times 100\%,\;\;i=1,\;\ldots ,\;N$$ (1.3)

where *N* is the number of voxels. We tested that normalizing by the mean BOLD signal during baseline (vs. mean across the whole time course) would produce no significant differences in CVR. Indeed, the baseline intensity did not differ significantly from the mean of the whole time course across subjects (7905 ± 130 vs. 7901 ± 130), implying that changes in BOLD signal induced by the BHs produce only a negligible perturbation of the baseline amplitude.

Following previous reports (Moia et al., 2020; Zvolanek et al., 2023), the optimal lags map was normalized to its median, yielding a spatially relative lag in each voxel, Lag_rel,i_:

$$Lag_{rel,i}(\text{sec})=Lag_{i}-M({\left\{Lag_{i}\right\}}_{i=1\ldots N}),\;\;i:1,\;\ldots ,\;N$$ (1.4)

where *M* represents the median operator.

Ultimately, the Lag_rel_ and CVR maps were thresholded to eliminate values below the 1^st^ percentile and above the 99^th^ or 90^th^ percentile, respectively, in accordance with the methodology established by Zvolanek et al. (2023), since these extreme values are deemed inadequately optimized.

### 2.4 Model comparisons

The models tested were compared by statistical analysis of the maps of F-values (model fit for the regressor of interest), CVR and relative optimal lag (Lag_rel_). Firstly, a region-of-interest (ROI) analysis was performed to compare different HRF delays/dispersions, along with different regressor types and convolution models. Based on this, a single optimal HRF delay/dispersion for the whole brain was selected and voxelwise analysis was then performed to further compare regressor types and convolution models across the brain.

#### 2.4.1 ROI analysis

For the ROI analysis, the average values of the voxelwise optimized F-value, CVR, Lag_rel_ maps in the GM and WM ROIs were computed. The effect of the HRF delay on the F-value was tested using a Friedman’s non-parametric test, for each of the two methods with convolution and each of the regressor types (Goss-Sampson, 2020). Posthoc pairwise comparisons were performed using Conover’s test and corrected for multiple comparisons using Bonferroni correction (p < 0.05). After choosing the appropriate delay based on the maximum F-value (the canonical delay of 6 s in every case—see results), the effects of the regressor type and convolution on the F-value, as well as on the CVR and Lag_rel_ values were tested using a Friedman’s non-parametric test (Goss-Sampson, 2020). Posthoc pairwise comparisons were performed using Conover’s test and corrected for the six multiple comparisons using Bonferroni correction (p < 0.008).

#### 2.4.2 Voxelwise analysis

For the voxelwise analysis, permutation testing was used to compare the two regression types for each of the convolution models (p-value corrected for the three multiple comparisons using Bonferroni correction, p < 0.017, three comparisons were made by comparing the two regressors for the same convolution model), and the three pairs of convolution models for each of the two regressor types (p-value corrected for the six multiple comparisons using Bonferroni correction, p < 0.008, six comparisons by comparing the three convolution models between themselves for the two regressor types), using FSL’s *Randomise* tool (Winkler et al., 2014). Because the number of multiple comparisons is different in each case, the corrected p-values assume different values.

## 3 Results

### 3.1 PetCO2 and block regressors

**Fig. 5.**
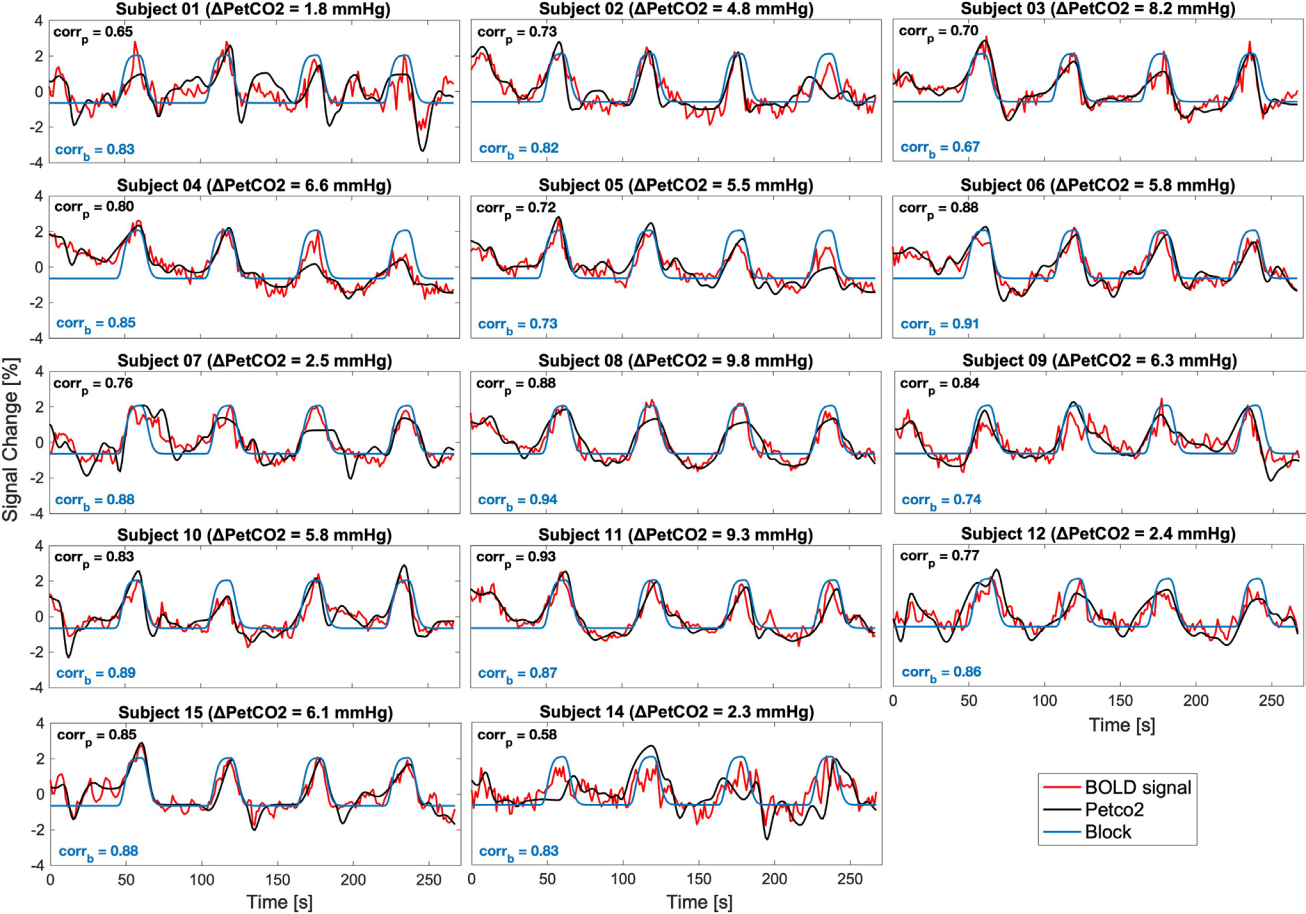
Individual responses to the breath-hold paradigm: average GM BOLD signals overlaid with the PetCO2 and Block regressors for all subjects. Both regressors were convolved with a single gamma HRF (delay = 6 s) and plotted considering the respective bulk lag that maximizes the correlation (with the value indicated in the figure by the corr_p,black_ and corr_b,blue_, for PetCO2 and block, respectively, and an average correlation across subjects equal to corr_p_ = 0.78 and corr_b_ = 0.83) between them and the average GM BOLD signal. The percent signal change was computed for the BOLD signal. The PetCO2 and the Block signals were normalized by demeaning the signal, dividing by the standard deviation, and multiplying by 100%.

The average GM BOLD signal that overlapped with the regressors type (Block, PetCO2) is plotted in Figure 5, considering the bulk lag that maximizes their correlation. The plots show that the BOLD signal is generally more correlated with PetCO2 regressors than Block regressors, as expected. Overall, the average GM BOLD signal appears to have adequate quality, indicating that the participants are effectively executing the task and that the signal is not suppressed by noise. The PetCO2 tracer is highly accurate, with the four BH blocks aligning with the results found for the average GM BOLD signal.

### 3.2 CVR and relative lag maps

**Fig. 6.**
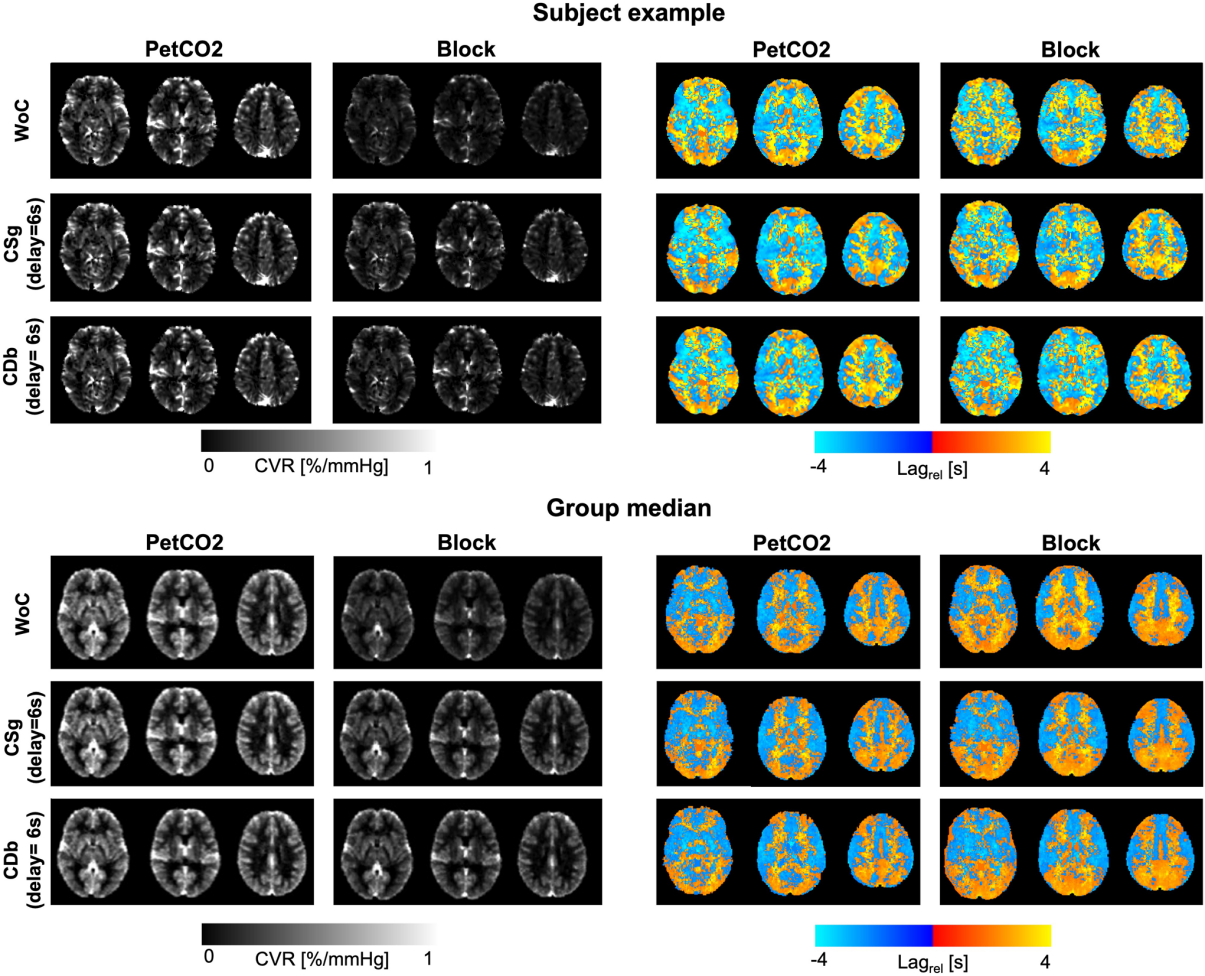
Subject example (top) and group median (bottom) maps of cerebrovascular reactivity (CVR) (left) and relative lag (Lag_rel_) obtained using the two regressor types (PetCO2 and Block) and the three convolution models (without convolution (WoC), convolution with single gamma (CSg), and convolution with double gamma (CDb), using the canonical delay of 6 s), for three representative slices in the MNI space.

Figure 6 presents a subject example and the group median maps of CVR and relative lag. The group maps exhibit a higher CVR for PetCO2 than for Block regressors, with values being more similar across the different convolution types. The combination of Block with WoC appears to yield the lowest CVR. In terms of relative lag, higher values, that is, a later response relatively to the median bulk lag, are obtained for Block and WM when compared with PetCO2 and GM respectively. Overall, the Lag_rel_ spatial distribution shows important differences between PetCO2 and Block regressors, with only small differences between convolution types.

Moreover, the distributions across subjects of the Lag_rel_ (for a HRF delay = 6 s) and CVR values can be found in Supplementary Figures S3 and S4, respectively. They allow us to verify that the ranges of lags tested were wide enough to take into account slower and faster responses in different brain tissues.

### 3.3 Effect of regressor type and convolution model

#### 3.3.1 ROI analysis

**Fig. 7.**
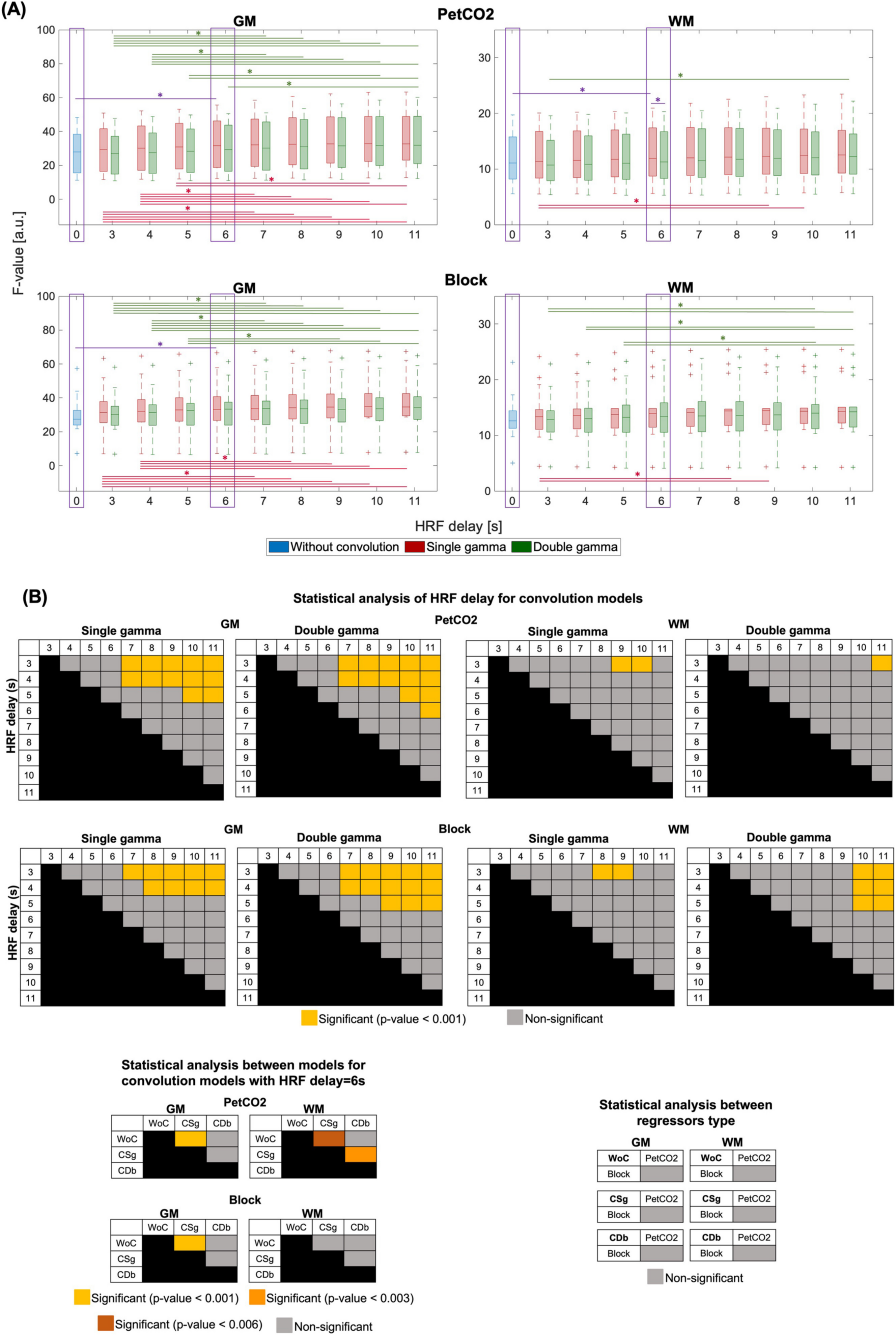
(A) ROI analysis of F-values, averaged across GM (left) and WM (right), for each of the models tested: two regressor types (PetCO2, top, and Block, bottom); three convolution models (without convolution (WoC), with convolution with single gamma (CSg), and with convolution with double gamma (CDb)); and different HRFs delays for the models with convolution (3-11 s) and (B) respective detailed statistical differences in model fitting. Boxplots represent the interquartile range of the distributions across subjects with + denoting outliers, and significant pairwise differences between convolution models are indicated with *. Purple box indicates the without HRF convolution and the convolution with an HRF delay = 6 s, for the convolution with single and double gamma.

The distributions across subjects of the mean F-value in GM and WM and respective detailed significant differences are displayed in Figure 7, for all the different models tested: regressor type (PetCO2, Block), convolution model (WoC, CSg, CDb), and HRF delay (3-11 s), with voxelwise lag optimization. Considering all the delays, a significant main effect of the delay (p-value < 0.001) is found for each convolution model and regressor. After choosing the appropriate delay (delay = 6 s), a main significant effect of the convolution model (p-value < 0.001) is found, as well as a significant interaction (p-value < 0.016) between the convolution model and the regressor. For both the PetCO2 and Block regressors, significant differences are found between WoC vs. CSg (delay 6 s), for all cases except for Block in WM, and between delays shorter than 6 s and delays longer than 7 s. Although significant differences between CSg and CDb are found only for PetCO2 in WM, CSg tends to yield higher F-values than CDb also in GM. Between the two regressor types (PetCO2 and Block), no significant differences are found. Based on the analysis of the F-values, we decided to keep the canonical HRF delay of 6 s only for subsequent analysis of CVR and Lag_rel_, since no significant improvements could be found with other delays.

**Fig. 8.**
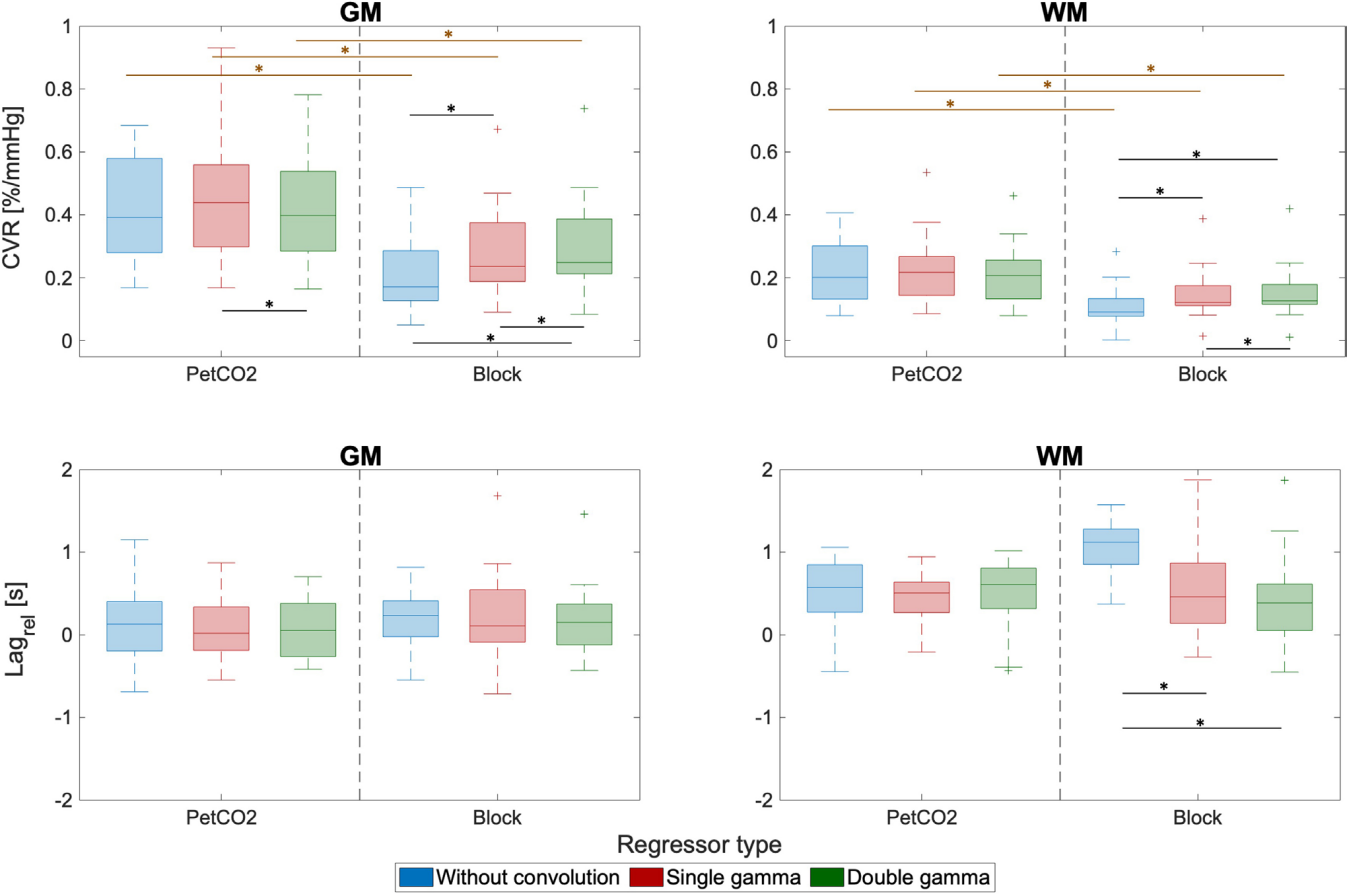
ROI analysis of CVR (first row) and Lag_rel_ (second row) values, averaged across GM (left) and WM (right), for the two regressor types (PetCO2 and Block) and the three convolution models (without convolution (WoC), with convolution with single gamma (CSg) and with convolution with double gamma (CDb), CDb)), with an HRF delay of 6 s for the convolution models). Boxplots represent the interquartile range of the distributions across subjects with + denoting outliers, and significant pairwise differences between convolution models are indicated with *. For CVR, all pairwise differences between regressor types (PetCO2 and Block) were statistically different (indicated in brown *).

The distributions across subjects of the average CVR and Lag_rel_ in GM and WM are displayed in Figure 8, for the different models tested: regressor type (PetCO2, Block) and convolution model (WoC, CSg, CDb).

For CVR, a significant main effect (p-value < 0.001) of the regressor type is found. The group median CVR values in GM are between 0.41–0.50%/mmHg and 0.2–0.30%/mmHg for PetCO2 and Block regressors, respectively, which are within the range reported in the literature (Moia et al., 2021), with CVR exhibiting statistically significant differences (p-value < 0.001) between the two regressor types for all convolution models. Moreover, a significant interaction (p-value < 0.001) between the convolution model and the regressor type is also found. For PetCO2, CSg yields the highest CVR in both GM and WM (but just significantly higher (p-value < 0.003) than CDb in GM). For Block, both convolution models, CSg and CDb, yield higher CVR values than models without convolution (WoC) (with significant differences (p-value < 0.001) between CDb and WoC in both GM and WM) and CDb also yield higher CVR than CSg (with significant differences (p-value < 0 .001) between CDb and CSg in both GM and WM).

For the Lag_rel_, a significant main effect (p-value < 0.005) is found for the convolution model. Specifically, when using the Block regressor, significantly longer lags are obtained for the models without convolution than for the two convolution models in WM.

#### 3.3.2 Voxelwise analysis

**Fig. 9.**
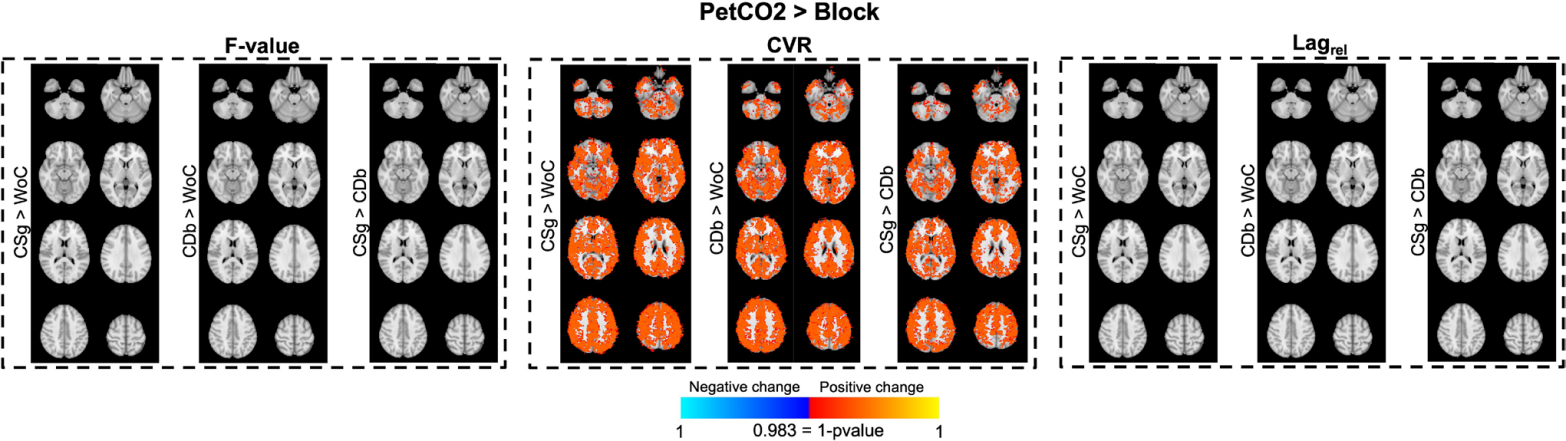
Voxelwise group-level permutation testing between regressors type (PetCO2 vs. Block) for the three convolution methods (without convolution (WoC), with convolution with single gamma (CSg), and with convolution with double gamma (CDb)), in terms of F-value (first column), CVR (second column), relative lag (Lag_rel_) (third column), and HRF delay (fourth column). Results are shown as 1-p-value maps for each comparison (both positive and negative changes) overlaid on MNI space T1-weighted image for four representative slices (colors representing significant differences placed over the MNI template). Permutation testing was performed using FSL’s Randomise, and the color bar represents the p-value with Family-Wise Error (FEW) correction; the p-value was thresholded after correction of the three multiple comparisons (p < 0.017, i.e., 1-0.017, p > 0.983).

**Fig. 10.**
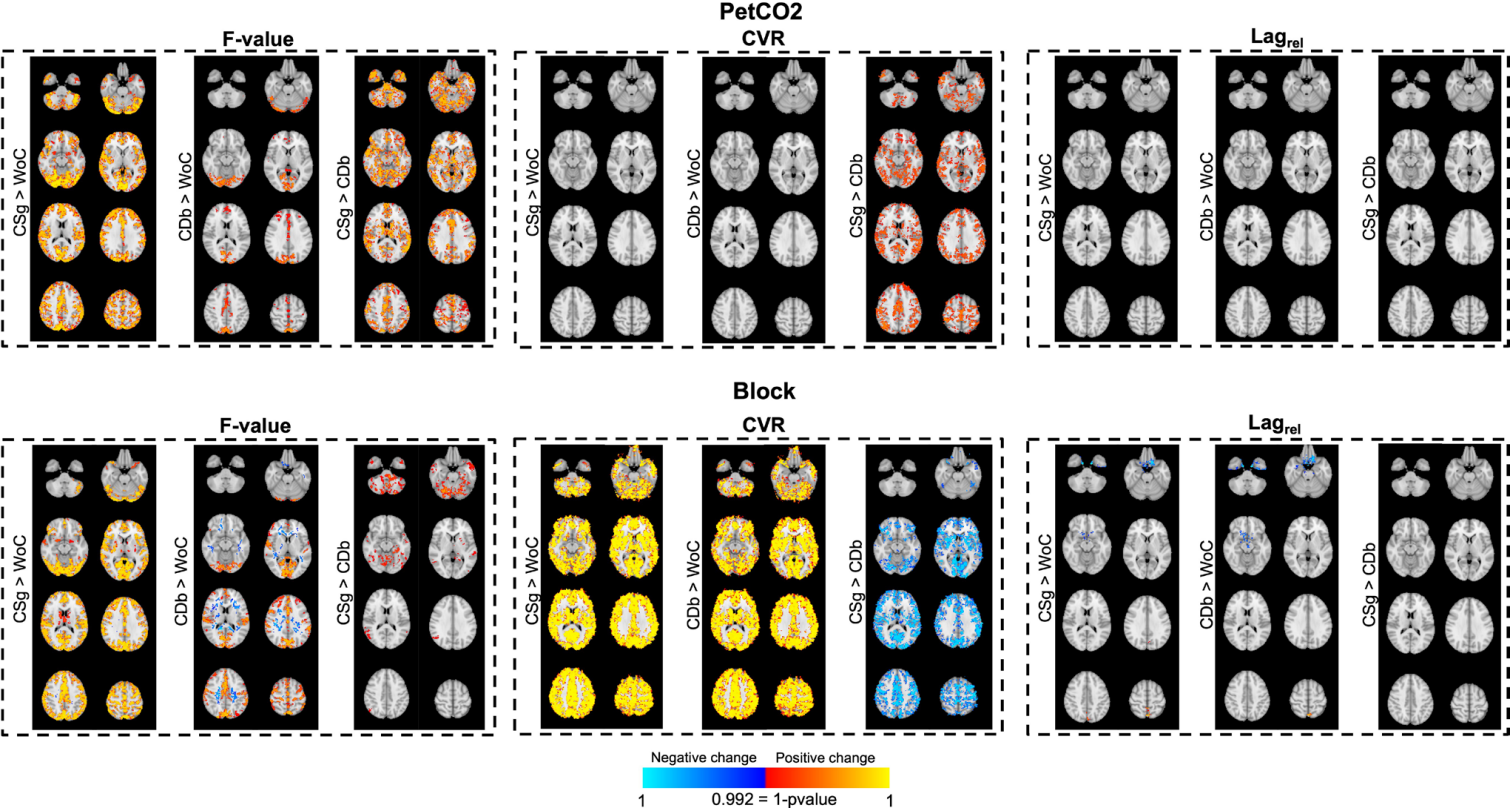
Voxelwise group-level permutation testing between convolution methods for the two regressor types (PetCO2, top, and Block, bottom) for the three convolution methods (without convolution (WoC), with convolution with single gamma (CSg), and with convolution with double gamma (CDb)), in terms of F-value (first column), CVR (second column), relative lag (Lag_rel_) (third column), and HRF delay (fourth column). Results are shown as 1-p-value maps for each comparison (both positive and negative changes) overlaid on MNI space T1-weighted image for four representative slices (colors representing significant differences placed over the MNI template). Permutation testing was performed using FSL’s Randomise, and the color bar represents the p-value with Family-Wise Error (FEW) correction; the p-value was thresholded after correction of the six multiple comparisons (p < 0.008, i.e., 1-0.008, p > 0.992).

The voxelwise comparisons between regressors type are presented in Figure 9 and the voxelwise comparisons between the convolution models in Figure 10. The voxelwise analysis showed significantly greater CVR across the whole brain for PetCO2 relative to Block regressors, for all convolution methods. No significant differences between regressor types are found for F-value or Lag_rel_. For PetCO2 regressors, F-value differences between without and with convolution are found across all GM (CSg > WoC and CDb > WoC). CVR values only differ between CSg vs. CDb, with CSg yielding higher values across GM. There are no significant differences in the Lag_rel_. For Block regressors, F-value differences are also observed between the convolution methods, with the two convolution methods outperforming no convolution across all of GM. CVR also differs between all methods, with CDb always yielding the greatest values across the whole GM. In terms of Lag_rel_, WoC differs significantly from CSg and CDb only in a few voxels in deep WM as well as CSg and CDb in a small occipital region.

### 3.4 EuskalIBUR dataset: cross-validation

The results obtained on the EuskalIBUR dataset are presented in the Supplementary Material. In general, the results obtained are largely consistent with the ones obtained with the IST dataset (as described on sections 3.1 to 3.3).

Figure S5 presents a subject example and the group median maps of CVR and Lag_rel_. As in the previous dataset, the group maps exhibit a higher CVR for PetCO2 than for Block regressors, with values being more similar across the different convolution types and again with the Block with WoC yielding the lowest CVR. In terms of relative lag, higher values, that is, a later response relatively to the median bulk lag, are observed for WM in comparison to GM. Comparatively lower values for PetCO2 are also observed in the Block, particularly evident in the Subject example.

The distributions across subjects of the mean F-value in GM and WM are displayed in Figure S6, for all the different models tested: regressor type (PetCO2, Block), convolution model (WoC, CSg, CDb), and HRF delay (3–11 s), with voxelwise lag optimization. Considering all the delays, a significant main effect (p-value < 0.001) of the delay is found for each convolution model and regressor. After choosing the appropriate delay (delay = 6 s), a main significant effect (p-value < 0.001) of the convolution model is found, as well as a significant interaction (p-value < 0.004) between the convolution model and the regressor. By comparing with the previous dataset and analysis, we found higher F-values for the CSg in all the cases (as observed before for the IST dataset), but with more differences between the HRF delays for the CDb, especially for delays higher than 9 s, although these delays do not seem fully appropriate as explained before. In terms of convolution methods, significant differences between CSg vs. WoC and CSg vs. CDb were found for both GM and WM, for the PetCO2 regressor, and between the three convolution methods for the Block regressor. Between the two regressor types (PetCO2 and Block), no significant differences are found, as also demonstrated previously.

The distributions across subjects of the average CVR and Lag_rel_ in GM and WM are displayed in Figure S7, for the different models tested: regressor type (PetCO2, Block) and convolution model (WoC, CSg, CDb). For CVR, a significant main effect (p-value < 0.001) of the regressor type was found. The group median CVR values in GM are between 0.17–0.20%/mmHg and 0.10–0.12%/mmHg for PetCO2 and Block regressors. Again, CVR exhibits statistically significant differences (p-value < 0.001) between the two regressor types for all convolution models. Moreover, a significant interaction between the convolution model and the regressor type is also found. For PetCO2, CSg yields the highest CVR (which is significantly higher (p-value < 0.002) than WoC and CDb in both GM and WM). For Block, both convolution models, CSg and CDb, yield higher CVR values than models without convolution (WoC) (with significant differences (p-value < 0.001) between CDb and WoC in both GM and WM) and CDb also yield higher CVR than CSg (with significant differences (p-value < 0.001) between CDb and CSg in WM).

For the Lag_rel_, no significant differences were found between convolution models or regressor type.

## 4 Discussion

We report the first systematic comparison of signal models for BH BOLD-fMRI data, when employing a voxelwise lag optimization. We found that PetCO2 regressors should be convolved with a single gamma HRF, while block regressors benefit from convolution with the double gamma HRF. Although PetCO2-based regressors yield generally greater CVR values than block-based regressors, the latter may be a better alternative in cases of poor CO_2_ recordings.

### 4.1 PetCO2 vs. block-based regressors

Although PetCO2 is, in general, the preferred regressor, greater patient compliance is needed for performing a BH task correctly and providing good-quality PetCO2 measurements. In fact, to obtain accurate CO_2_ measurements using a nasal cannula, patients must breathe out through the nose and not through the mouth and the nasal cannula must not be displaced during the experiment. Unfortunately, such problems often arise when studying less compliant subjects as it happens in certain patient populations. To assess this issue, we present additional data, collected from four migraine patients in the scope of the same project from which the data used in this study were taken in Supplementary Figure S8. In these cases, poor CO_2_ recordings led to PetCO2 regressors that were not well correlated with the measured BOLD signal (r < 0.5). In these cases, the subjects seemed to perform the BH task correctly (given the observed variations in the BOLD signal, following the task). Still, the CO_2_ recordings do not correctly detect the associated PetCO2 increases. As indicated before, this is a known problem, probably related to the subject’s non-compliance with breathing out through the nose or to a misplacement of the nose cannula, leading to poor CO_2_ measurements. In such cases where PetCO2 is not a reliable measure, using a Block paradigm instead is recommended and, indeed, yielded better results than a poor PetCO2 regressor (for example, in one of these subjects (represented in Supplementary Fig. S8), the estimated average CVR for a poor PetCO2 was 0.03 %/mmHg and for Block it was 0.20 %/mmHg). It should also be noted that, despite the difference in the estimated CVR values, the BOLD signal variance explained by the two types of regressors is not significantly different. Several studies also used a ramp paradigm rather than a block (Bright & Murphy, 2013; Evanoff et al., 2020). In this work, a ramp was included as a regressor type in a preliminary analysis (see Supplementary Fig. S9); however, because the block and ramp explained similar amounts of signal variance, the ramp was excluded from subsequent analysis. Importantly, if PetCO2 and Block regressors are used concurrently in the same study for different participants, the regressor type should be included as a confound in the statistical model for a more accurate presentation of the results and to eliminate potential bias resulting from this.

When a reliable PetCO2 signal cannot be recorded, another option to consider is to use a respiratory belt to measure the chest expansion during a BH task and retrieve the respiration volume per time (RVT) as a proxy of PetCO2 changes as investigated by Zvolanek et al. (2023), but not addressed in this study. The BOLD signal model is then obtained by convolving the RVT with a respiratory response function (RRF) (Birn et al., 2008; Zvolanek et al., 2023), so testing the effect of using different RRFs could be an interesting work to develop. Another approach that does not require any additional measurements is to use a Fourier model assuming a periodic signal change in response to a sequence of BH periods (Lipp et al., 2015; Murphy et al., 2011; Pinto et al., 2016). In this case, an appropriate number of harmonics may be added to the task frequency to adjust the model to the dynamics of the BOLD response. However, the adequacy of such an approach strongly depends on the periodicity of the paradigm, which may vary depending on the duration of the BH and normal breathing periods.

### 4.2 Effect of convolution and HRF shape

We found that convolving PetCO2 with a single gamma HRF yielded a significant higher F-value as well as a higher CVR than convolving with the commonly used double gamma HRF. Even for a block-based regressor, although a single gamma HRF did not perform as well as a double gamma HRF, it did significantly better than no convolution. The observed differences in the impact of the convolution model between PetCO2 and block models could be explained by the fact that PetCO2 fluctuations more closely reflect the dynamics of the BH task and are, therefore, more closely linked to the elicited blood flow and associated BOLD increase (Golestani et al., 2015). As a result, while a block model without convolution is unsuitable and can benefit from the more complex double gamma HRF relative to the single gamma HRF, a PetCO2 model may be convolved with a single gamma HRF. In addition, the presence of negative amplitudes in PetCO2 could be artifactual, and the double gamma will only accentuate this, yielding worse findings than the single gamma. Consistently with our results, the function retrieved by Golestani et al. (2015) by deconvolving the PetCO2 time course from resting-state BOLD fMRI data, was a single gamma. Nevertheless, in this study, the authors found that a double gamma could be a better fit for datasets with a higher TR. Since the TR values of both datasets we analyzed (1.26 and 1.50 s) are quite typical in current fMRI acquisitions, we believe that our results should be generalizable to most studies.

It should be noted that the voxelwise optimization of the lag, by fitting multiple GLM’s and selecting the one yielding the best model fit in each voxel, leads to a multiple comparisons problem, implying an increased model complexity. Correcting for multiple comparisons would impact the significance of the model fit, and hence its parameter estimates. However, we did not restrict our CVR maps to the voxels exhibiting significant model fits, that is, they were not obtained through statistical inference (and corresponding thresholding), we just thresholded our CVR and Lag_rel_ maps to excluded outliers. The procedure of correcting the CVR maps through statistical inference is not consistent in the literature, where some works correct the statistical maps for multiple comparisons (Moia et al., 2020, 2021; Stickland et al., 2021), while others only apply an amplitude threshold to exclude higher or lower values (Zvolanek et al., 2023).

### 4.3 Effect of HRF delay and dispersion

Our initial analysis revealed that while conducting a voxelwise HRF delay optimization yields a higher overall F-value, this F-value does not accurately represent the model and creates a misleading impression of optimization. In some subjects, this value, in fact, decreases when compared to the canonical HRF delay. Moreover, an interaction with lag leads the outcome of one to skew the results of the other. These findings are consistent with previous reports indicating that the HRF delay interacts with the time lag toward describing the tissue’s response to the BH and associated PetCO2 increase (K. Chen et al., 2021; Yao et al., 2021).

### 4.4 Relation with previous model comparisons

Our model comparison results are consistent to the extent possible with the relevant literature. Only one previous study systematically compared models of BOLD responses to BH tasks in terms of the regressor type as well as the HRF convolution (Murphy et al., 2011). They found that convolution with a canonical HRF explained significantly more variance than no convolution, as detected in our case when performing a voxelwise HRF delay optimization.

Moreover, Murphy et al. (2011) reported a significant increase in the variance explained when a temporal derivative was added, both when using PetCO2 and block regressors, which we did not observe (preliminary tests, results shown in Supplementary Fig. S10). Again because of our voxelwise HRF delay and lag optimization, the temporal derivative should have a smaller impact in our case. Although including the termporal derivative allows accounting for some lag variations across the brain, it is less effective than voxelwise lag optimization. Murphy et al. (2011) considered only convolution with the double gamma canonical HRF, and we can therefore not directly compare our results for the single gamma HRF with theirs.

It should be noted that we decided to consider variations of the canonical HRF because this has been the most common practice in the analysis of BH BOLD data. However, it is known that the physiological response in a BH task differs from the neuronal response in brain activation tasks as well as from other respiratory tasks, and different HRF shapes could, therefore, be considered. This was previously done for paced hyperventilation tasks, by estimating the response function to PetCO2 fluctuations, and a function resembling a double gamma with a more pronounced undershoot was found (Vogt et al., 2011). Another study considered a wide range of candidate HRFs to model the BOLD response to a CO_2_ challenge (Yao et al., 2021) and reported that different HRFs should be employed in the same model to appropriately map CVR. As a result, while the single gamma HRF can be an acceptable model in general, there may be some regional differences.

### 4.5 CVR and relative lag maps

In general, for all models tested, the CVR and relative lag maps obtained in our study are consistent with the literature employing voxelwise lag optimization through a lagged GLM analysis of BH BOLD-fMRI data (Moia et al., 2020, 2021; Stickland et al., 2021; Zvolanek et al., 2023). In terms of the spatial distribution, we observe a lower CVR value and a greater lag, that is, a smaller and later response, for WM compared with GM. In terms of the GM and WM average values, CVR and relative lag are also within the range of values previously reported (group average CVR values around 0.49 %/mmHg for GM and 0.21 %/mmHg to WM for all the convolution models tested; and relative lags around -0.2 s and 0.2 s, for GM and WM, respectively, using PetCO2 as the regressor) (Moia et al., 2021). Overall, the consistency of the CVR and lag values we obtained indicates that our model optimization procedure is not producing artifactual signal models. Indeed, we carefully restricted our model space to physiologically meaningful models, in terms of the response shape as well as its delay/dispersion and lag (Liao et al., 2002; Lindquist & Wager, 2007). Because of this, and since we have a BH task rather than a CO_2_ gas challenge, we considered some delays evaluated in Yao et al. (2021) or Shams et al. (2023) but using less wide range, since we did not go beyond an HRF delay of 11 s, nor did consider other combinations of the different factors.

Although the median values of voxelwise optimal lags are in general very close to the bulk lag, retrieved from the cross-correlation between the GM average BOLD time series and PetCO2, significant regional differences are observed as previously reported (Moia et al., 2020, 2021; Stickland et al., 2021; Zvolanek et al., 2023). The observation of such variability in the hemodynamic delays supports the need of voxelwise optimization to obtain more accurate CVR maps. Nonetheless, in this study and as done in most other studies (Moia et al., 2020, 2021; Stickland et al., 2021; Zvolanek et al., 2023), the bulk lag was calculated with respect to GM voxels. To obtain better results in WM, estimating the bulk lag for WM could be an additional optimization step to consider. This assumption, combined with the fact that the HRF shape is mostly optimized and reported for GM areas such as visual or motor areas, the WM is also more affected by physiological fluctuations caused by cardiac and respiratory variation (Li et al., 2022), and WM voxels exhibit a lower BOLD signal change than GM voxels, could explain the less significant results observed in WM (as shown in Fig. 10).

### 4.6 EuskalIBUR dataset: cross-validation

The investigation across an independent dataset revealed that the results were largely reproduced, indicating their generalizability. In both datasets, CSg convolution yielded higher CVR with PetCO2 regressors while CDb convolution yielded higher CVR with Block regressors. Some minor discrepancies could be attributed to the larger BH duration in the EuskalIBUR dataset (15 s vs. 20 s), which may have an impact on BOLD response shape. For longer or shorter BH durations than this, the findings may differ and further testing should be performed. Nevertheless, the range of durations between the two datasets evaluated here is the recommended for an optimal BH task (Pinto et al., 2021). Furthermore, (Moia et al., 2021)—EuskalIBUR dataset used the optimal combination of the four echoes which can aid in the reduction of signal dropout and thermal noise.

### 4.7 Limitations

We are using a nasal cannula to monitor the patient’s inhalation and exhalation. However, if the patient breathes not only through the nose but also through the mouth, the PetCO2 tracer may not be accurate (as evidenced by Supplementary Figure S8, where the average GM BOLD signal indicates that the participants appear to be performing the task, although the PetCO2 tracer is not very suitable). This issue can be resolved by utilizing a face mask covering both nose and mouth, albeit causing additional experimental complexity and patient discomfort. In this study, the data are mostly unaffected by it, as the PetCO2 tracers exhibit high quality and align with the average GM BOLD signal, indicating that the subjects are executing the task and that the PetCO2 tracer is consistent with this performance.

One potential limitation of our results is that they pertain specifically to the BH protocol we employed, which consisted of end-expiration breath-holding. In fact, it has been shown that BOLD responses are significantly different when executing BH after expiration relative to inspiration (Pinto et al., 2021). Likewise, the BOLD response to other CVR-inducing stimuli, such as other breathing tasks like cued deep breathing or gas challenges, is likely to differ from the one to a BH task, and specific studies need to be performed to optimize modeling approaches in those cases (e.g., Yao et al., 2021).

Another limitation of our study is that it was conducted in a healthy population; while it is a methodological study from which we can draw some conclusions, it is also true that in pathologies or even healthy aging, hemodynamic measures of the brain, such as hemodynamic delay and CVR optimal lag, can change (Krishnamurthy et al., 2021; Pinto et al., 2021; Zvolanek et al., 2023). As a result, extra care should be applied when performing investigations in illnesses where hemodynamic delays are likely to be significantly different from the standard. Complementary to this limitation is the fact that the IST dataset analyzed here was acquired from a cohort of women only. Although some papers reported CVR differences between males and females (D. Y. Chen et al., 2024; Koep et al., 2022), there is no evidence of gender differences in the shape of the BOLD response to BH tasks. Nevertheless, future studies with more diverse cohorts are needed to clarify whether the BOLD signal modeling approach may be sex-specific.

Despite the report by Ravi et al. (2016) showing that long echo times (such as the one used in our study, TE = 30 ms) may result in negative CVR artifacts, most studies employing BOLD-fMRI for CVR mapping, nevertheless, use echo times similar to ours (or even longer), such as Bright and Murphy (2013), Murphy et al. (2011) or Sleight et al. (2023), who used TE = 35 ms. Our results should, therefore, be relevant to most existing studies, but future studies should consider further optimizing echo times.

## 5 Conclusion

Our study demonstrates that better measures of CVR can be obtained by applying optimized BOLD signal models. For an end-expiration BH-BOLD fMRI paradigm, we recommend using the PetCO2 signal convolved with a single-gamma HRF, with voxelwise optimization of lag. Since the BOLD response shape differs for BH tasks performed at the end of an expiration versus an inspiration (Vogt et al., 2011), we did not consider such variation and further studies will need to be conducted to find the optimal modeling approach for end-inspiration paradigms. Nevertheless, we believe that models of end-expiration tasks should be most impactful, given that this has been the recommended task in a recent guidelines publication (Pinto et al., 2021). When good quality CO_2_ recordings are not available, convolution of a block design with the canonical double-gamma HRF is a suitable alternative.

## Supplementary Material

Supplementary Material

## Data Availability

Available upon a formal data sharing agreement.
