# Supplementary material for: Cerebrovascular reactivity mapping using breath-hold BOLD-fMRI: Comparison of signal models combined with voxelwise lag optimization
Source: Imaging Neurosci (Camb). 2025 Jul 14;3:IMAG.a.80. doi: 10.1162/IMAG.a.80 (PMC12330853; doi:10.1162/IMAG.a.80)
Supplement: Supplementary Material [file IMAG.a.80_supp.pdf]

SUPPLEMENTARY MATERIAL

Supplementary Figures S1-S10.

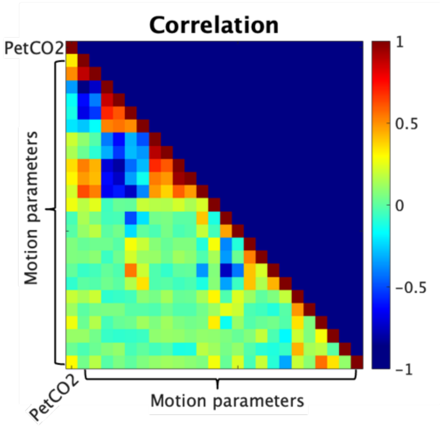

Figure S1: Illustrative example of the correlation matrix between the regressors in the GLM, for the PetCO2 regressor convolved with a single-gamma HRF, with HRF delay=6s, and the bulk lag (first row and column), and the 24 motion parameters (remaining rows and columns).

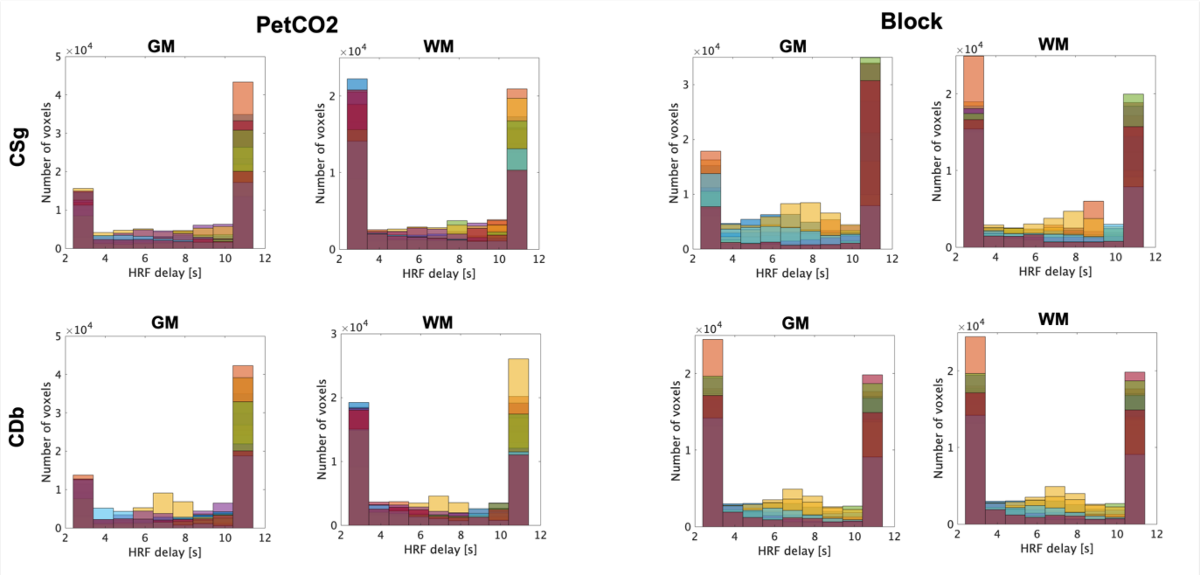

Figure S2: Distributions of the HRF delay, across GM and WM regions of the maps of all subjects, for the two convolution models (convolution with a single gamma (CSg) and convolution with a double gamma (CDb)) and the two regressor types (PetCO2 and Block).

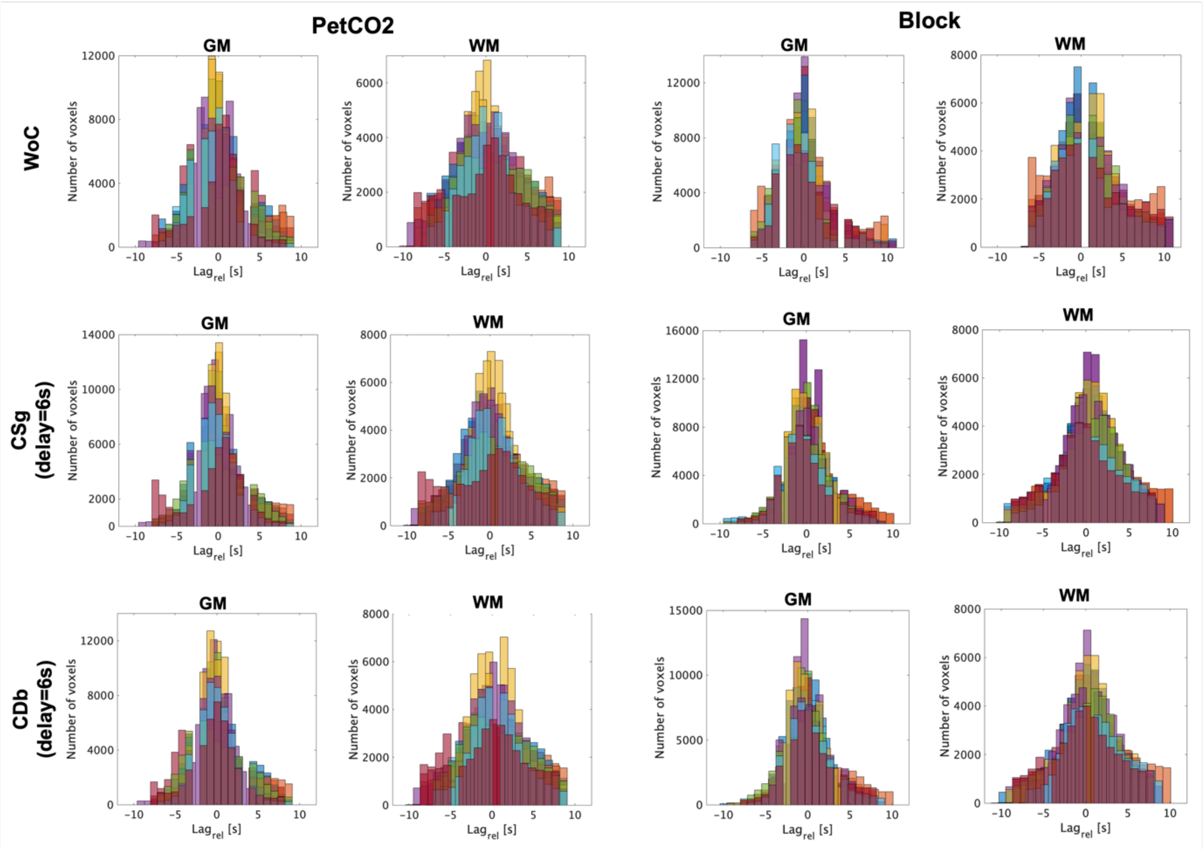

Figure S3: Distributions of the relative lag across GM and WM regions of the maps of all subjects, for the three convolution models (without convolution (WoC), convolution with single gamma (CSg) and convolution with double gamma (CDb), using the canonical delay of 6s) and the two regressor types (PetCO2 and Block).

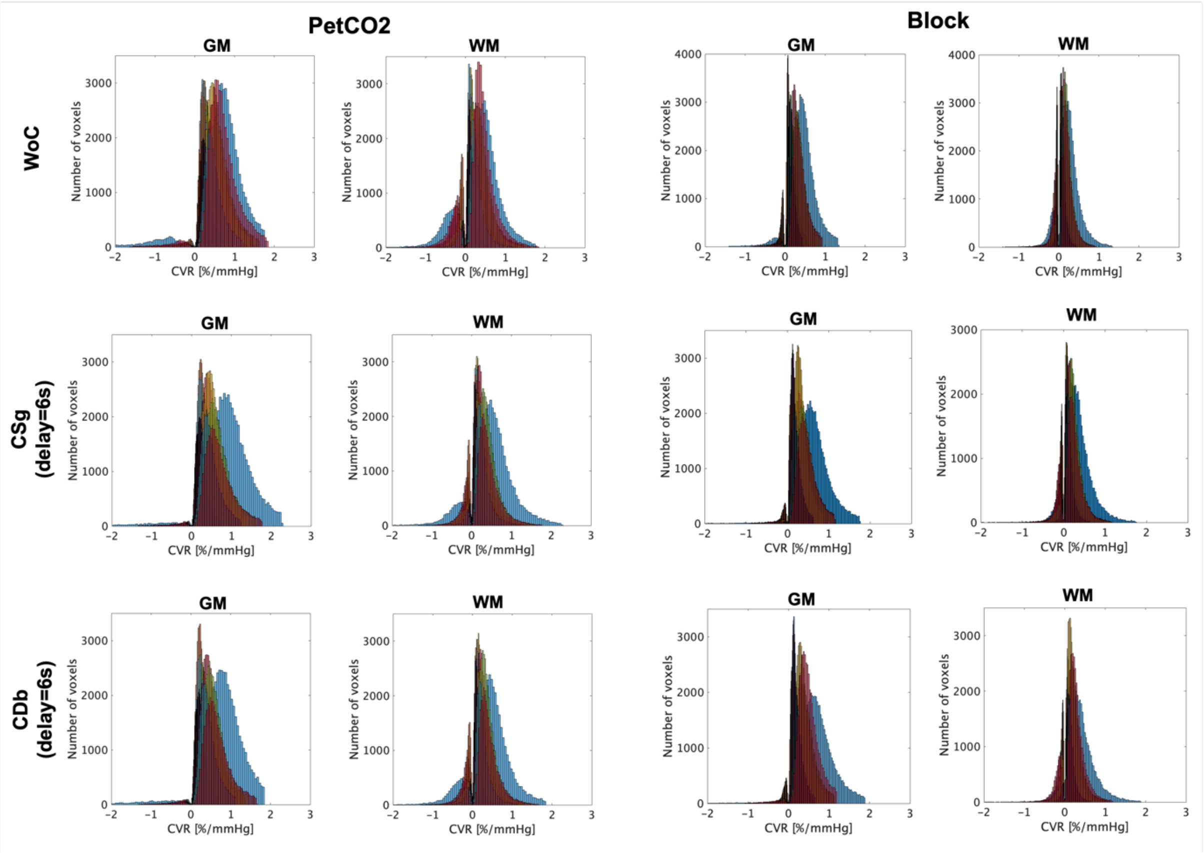

Figure S4: Distributions of the CVR across GM and WM regions of the maps of all subjects, for the three convolution models (without convolution (WoC), convolution with single gamma (CSg) and convolution with double gamma (CDb), using the canonical delay of 6s) and the two regressor types (PetCO2 and Block).

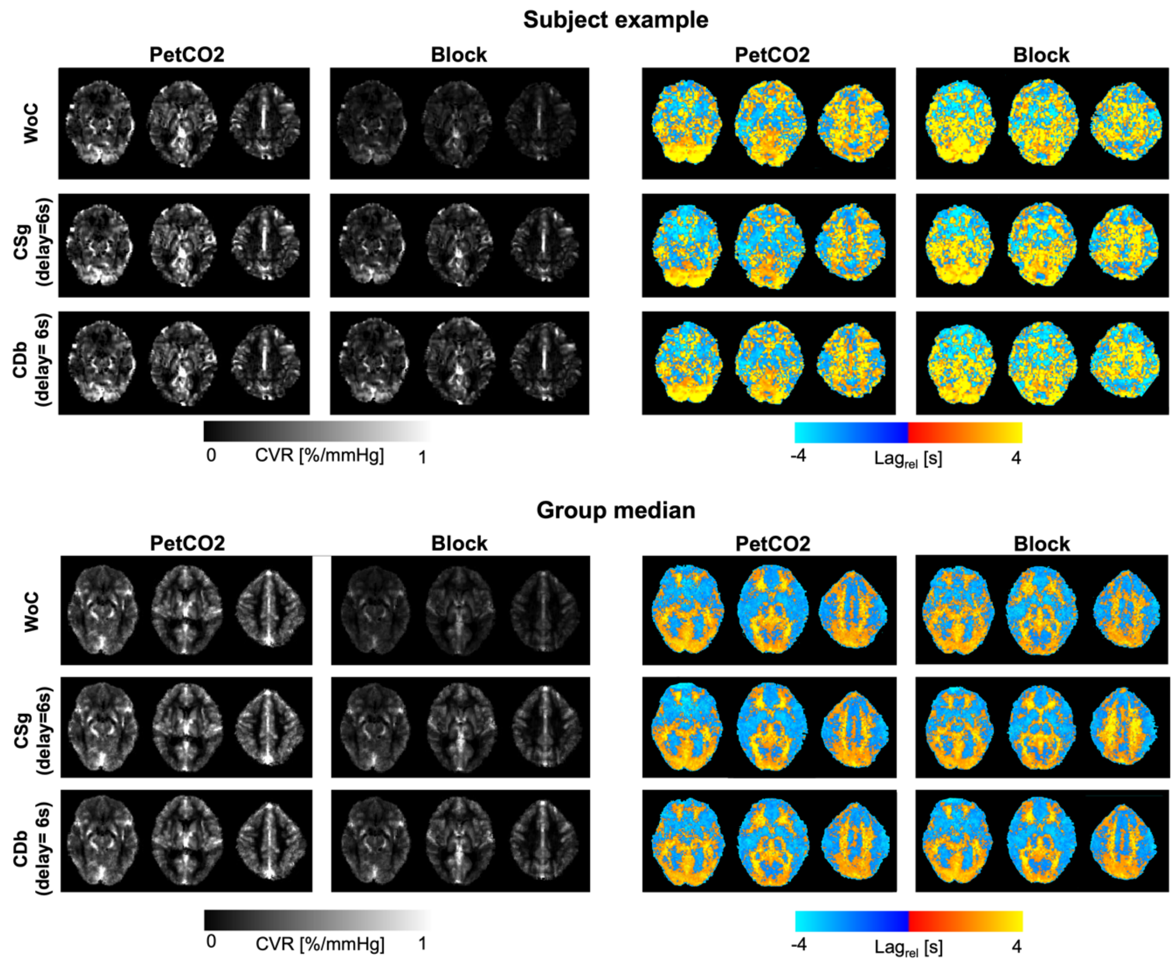

Figure S5: Subject example (top) and group median (bottom) maps of cerebrovascular reactivity (CVR) (left) and relative lag (Lag$_{rel}$) *obtained using the two regressor types (PetCO2 and Block) and the three convolution models (without convolution (WoC), convolution with single gamma (CSg) and convolution with double gamma (CDb), using the canonical delay of 6s), for three representative slices in the MNI space.*

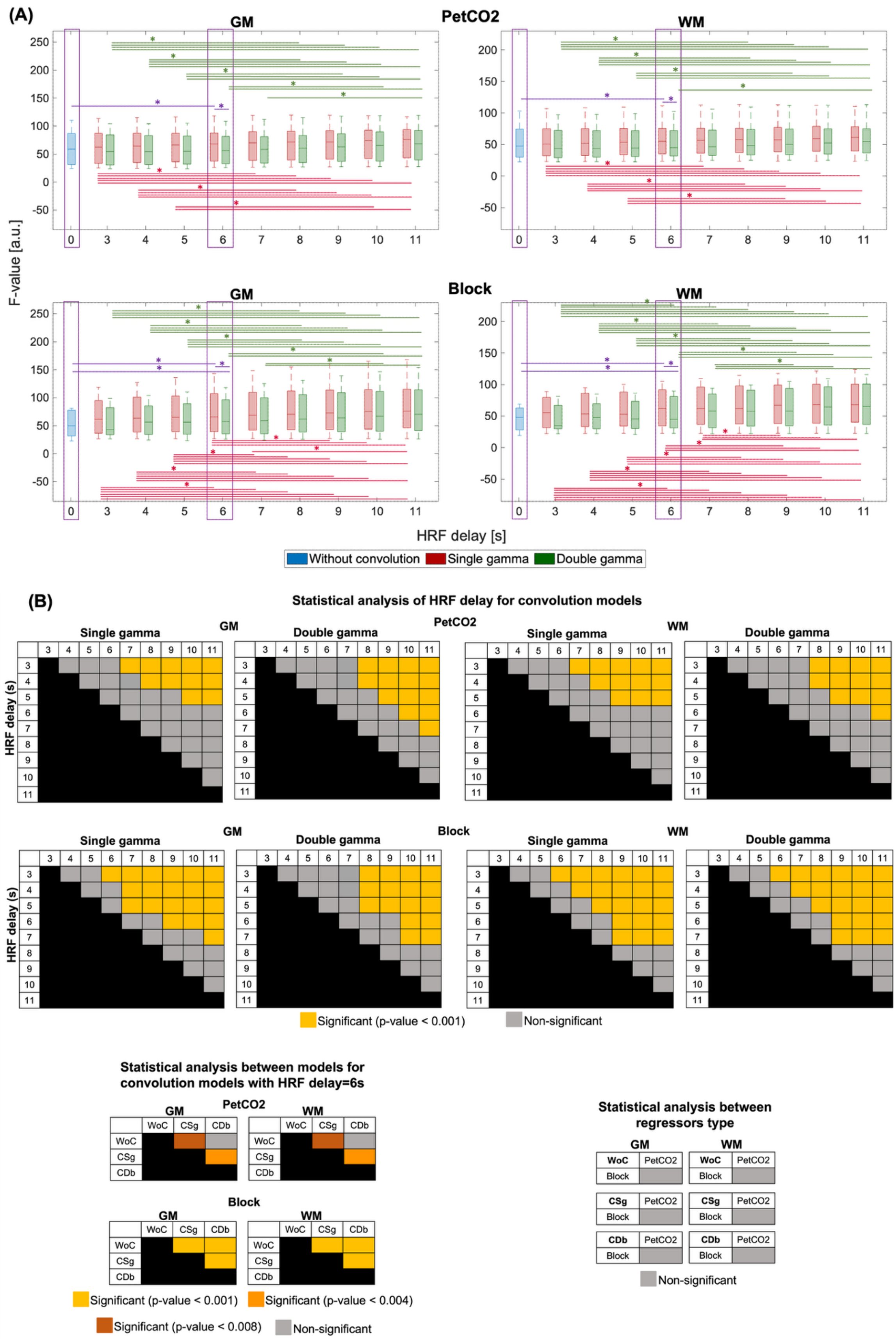

(A)
PetCO2
GM
WM
Block
F-value [a.u.]
HRF delay [s]
Without convolution
Single gamma
Double gamma
(B)
Statistical analysis of HRF delay for convolution models
Single gamma
Double gamma
HRF delay (s)
Significant (p-value < 0.001)
Non-significant
Statistical analysis between models for convolution models with HRF delay=6s
WoC
CSg
CDb
Significant (p-value < 0.004)
Significant (p-value < 0.008)
Statistical analysis between regressors type

Figure S6: (A) ROI analysis of F-values, averaged across GM (left) and WM (right), for each of the models tested: two regressor types (PetCO2, top, and Block, bottom); three convolution models (without convolution (WoC), with convolution with single gamma (CSg) and with convolution with double gamma (CDb)); and different HRFs delays for the models with convolution (3-11s) and (B) respective detailed statistical differences in model fitting. *Boxplots represent the interquartile range of the distributions across subjects and significant pairwise differences between convolution models are indicated with *. Purple box indicates the without HRF convolution and the convolution with a HRF delay=6s, for the convolution with single and double gamma.*

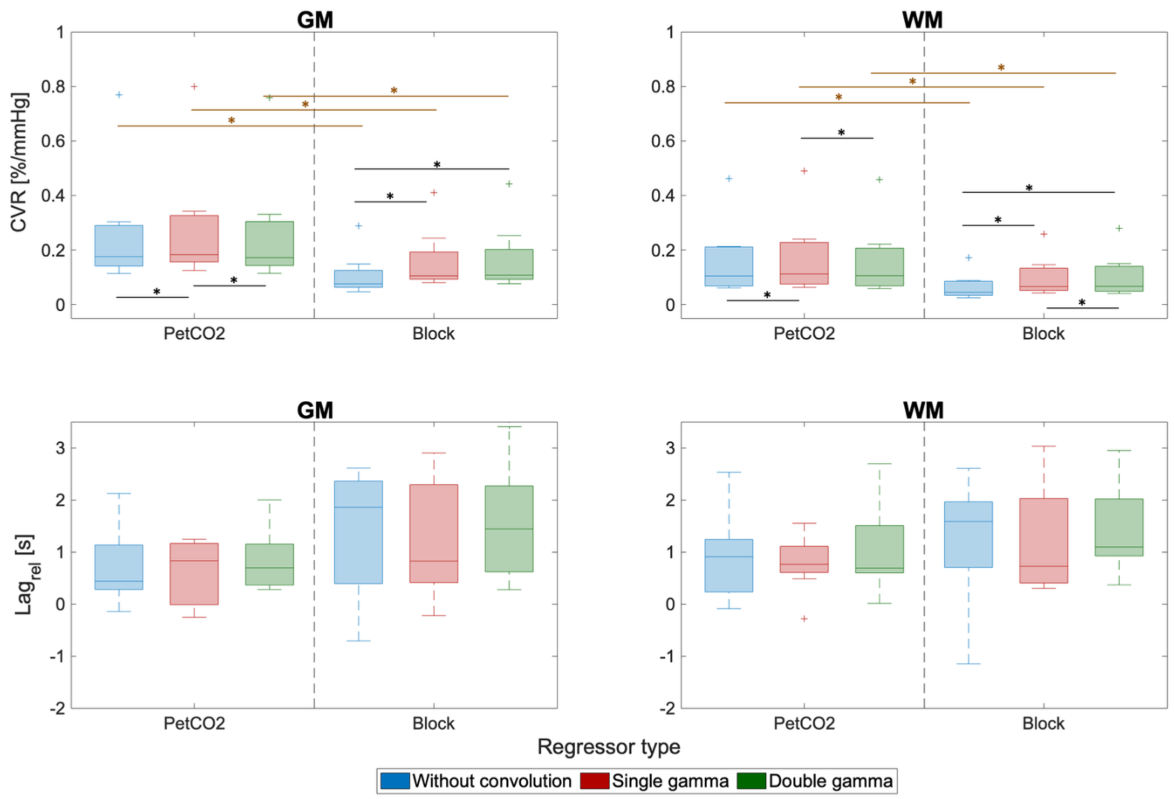

Figure S7: ROI analysis of CVR (first row) and Lag$_{rel}$ (second row) values, averaged across GM (left) and WM (right)*, for the two regressor types (PetCO2 and Block) and the three convolution models (without convolution (WoC), with convolution with single gamma (CSg) and with convolution with double gamma (CDb), CDb)), with an HRF delay of 6s for the convolution models). Boxplots represent the interquartile range of the distributions across subjects and significant pairwise differences between convolution models are indicated with *. For CVR, all pairwise differences between regressor types (PetCO2 and Block) were statistically different (indicated in brown *).*

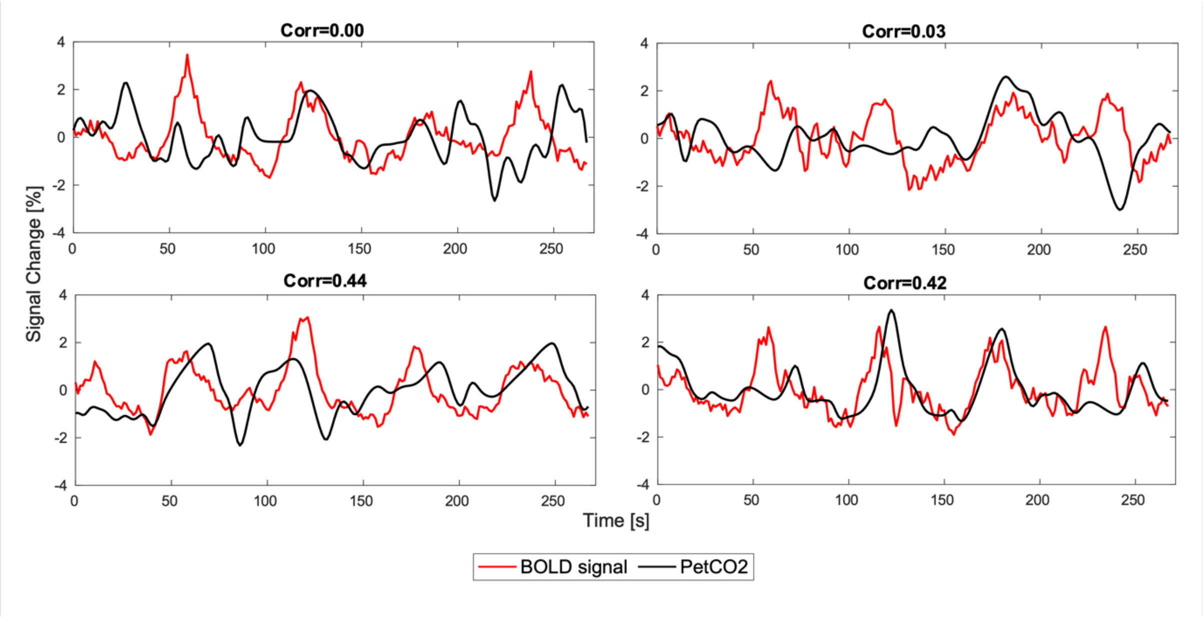

Figure S8: Examples of poor CO_2 traces in the breath-hold task, for four migraine patients (one in each panel): PetCO2 signal overlaid with the average GM BOLD signal. *A correlation (Corr) of 0.5 between the two signals was defined as the minimum value to admit the CO_2 trace as good quality.*

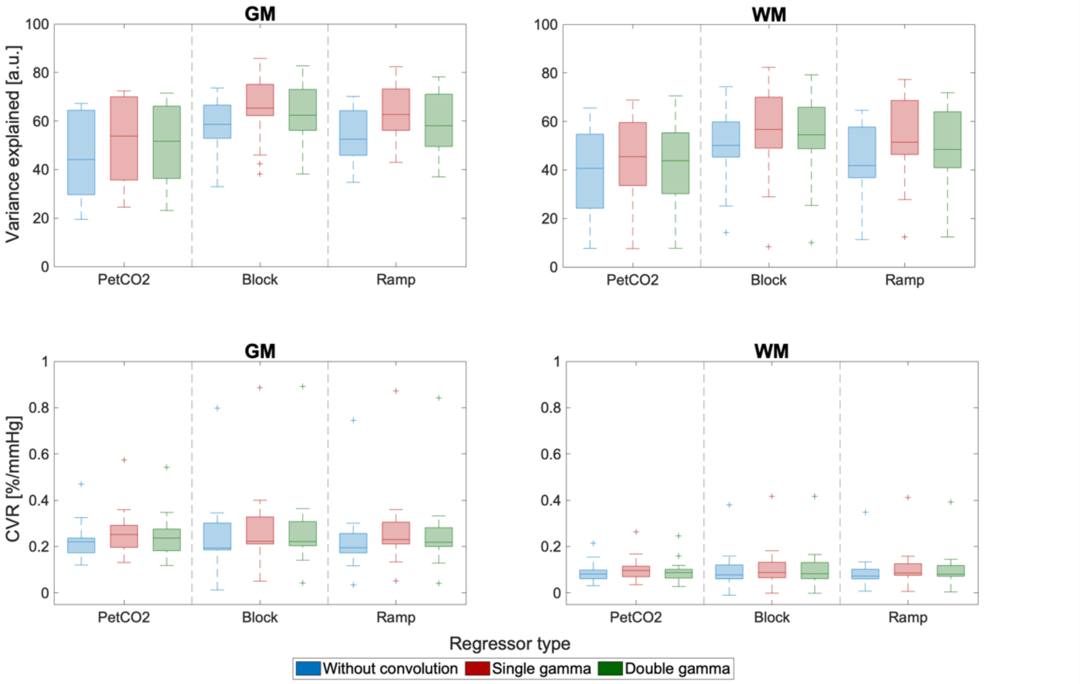

Figure S9: ROI preliminary analysis of the variance explained (first row) and CVR (second row) values, obtained for the average GM (left) and WM (right) BOLD timeseries*, for three regressor types (PetCO2, Block and Ramp) and the three convolution models (without convolution (WoC), with convolution with single gamma (CSg) and with*

convolution with double gamma (CDb), CDb)), with an HRF delay of 6s for the convolution models). Boxplots represent the interquartile range of the distributions across subjects.

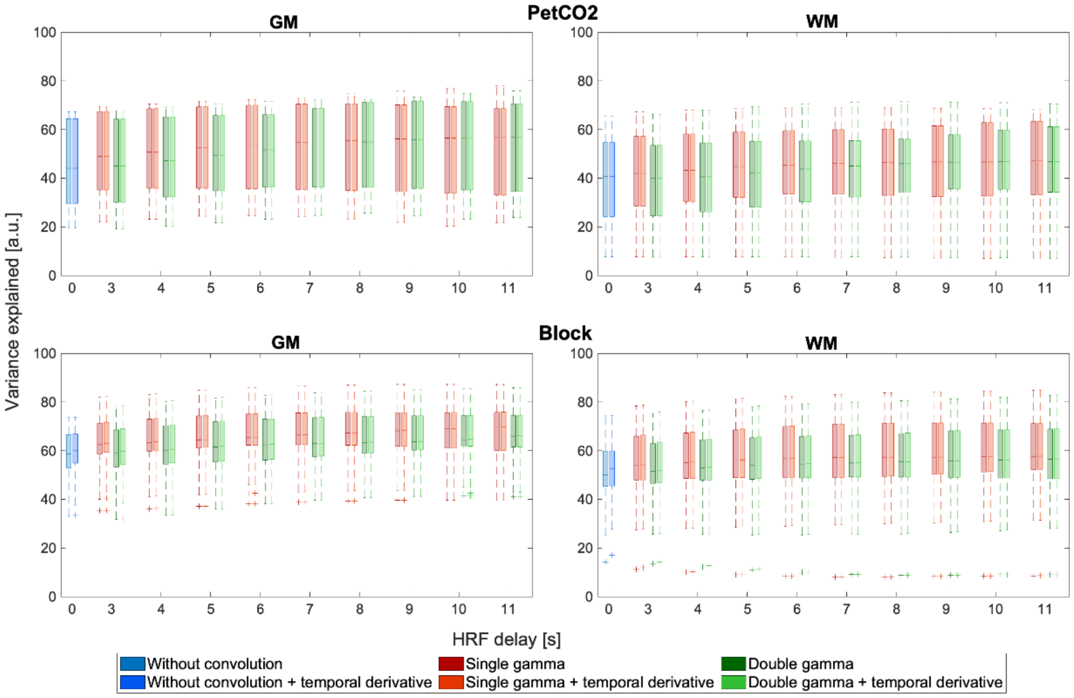

Figure S10: ROI preliminary analysis of the variance explained, obtained for the average GM (left) and WM (right) BOLD timeseries, for each of the models tested: two regressor types (PetCO2, top, and Block, bottom); three convolution models (without convolution (WoC), with convolution with single gamma (CSg) and with convolution with double gamma (CDb)); and different HRFs delays for the models with convolution (3-11s) with and without temporal derivative.
